# ATP6AP1 promotes cell proliferation and tamoxifen resistance in luminal breast cancer by inducing autophagy

**DOI:** 10.1038/s41419-025-07534-y

**Published:** 2025-03-25

**Authors:** Zhengwei Yan, Aidi Huang, Dongwen Ma, Chenao Hong, Shengmiao Zhang, Luling He, Hai Rao, Shiwen Luo

**Affiliations:** 1 Center for Experimental Medicine, The First Affiliated Hospital of Nanchang University; The MOE Basic Research and Innovation Center for the Targeted Therapeutics of Solid Tumors; Jiangxi Medical College, Nanchang University, Nanchang, Jiangxi 330006 China; 2 Department of Biochemistry, School of Medicine, Key University Laboratory of Metabolism and Health of Guangdong, Southern University of Science and Technology, Shenzhen, 518055 China; 3 Department of Pathology and Institute of Molecular Pathology, Jiangxi Provincial Key Laboratory for Precision Pathology and Intelligent Diagnosis, The First Affiliated Hospital of Nanchang University, Nanchang, Jiangxi 330006 China

**Keywords:** Breast cancer, Autophagy, Cell growth, Mechanisms of disease, Tumour biomarkers

## Abstract

Autophagy is a highly conserved cellular process essential for maintaining cellular homeostasis and influencing cancer development. Lysosomal acidification and autophagosome-lysosome fusion are two important steps of autophagy degradation that are tightly regulated. Although many key proteins that regulate these two events have been identified, the effector proteins that co-regulate both steps remain to be explored. ATP6AP1, an accessory subunit of V-ATPase, plays a critical role in the assembly and regulation of V-ATPase. However, the function of ATP6AP1 in autophagy remains unknown, and the role of ATP6AP1 in cancer is still poorly understood. In this study, we found that ATP6AP1 is overexpressed in luminal breast cancer tissues and promotes the proliferation and tamoxifen resistance of luminal breast cancer cells both in vitro and in vivo. We also observed that high ATP6AP1 expression correlates with poor overall patient survival. Our research further revealed that ATP6AP1 enhances tamoxifen resistance by activating autophagy. Mechanistically, ATP6AP1 promotes autophagy by regulating both lysosomal acidification and autophagosome-lysosome fusion. Remarkably, ATP6AP1 induces lysosomal acidification through the regulation of V-ATPase assembly and facilitates autophagosome-lysosome fusion by enhancing the interaction between Rab7 and the HOPS complex. Together, our studies identify ATP6AP1 as a crucial regulator of autophagy, potentially serving as a valuable prognostic marker or therapeutic target in human luminal breast cancer.

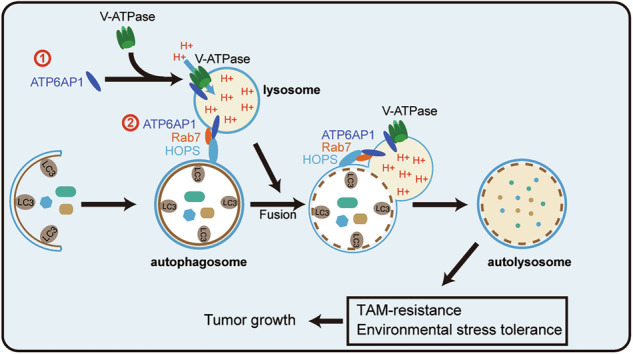

## Introduction

Macroautophagy (hereafter termed autophagy) is a crucial catabolic process that degrades unnecessary or dysfunctional proteins, macromolecules, and organelles through lysosomes. Autophagy delivers cytoplasmic cargo to the lysosome for destruction through the intermediary of a double membrane-bound vesicle, termed an autophagosome, which fuses with the lysosome to form an autolysosome [1, 2]. When lysosomal biogenesis or delivery is impaired, endocytic and autophagic cargo remain trapped in late endosomes/autophagosomes and are inefficiently degraded, leading to various pathological conditions, such as neurodegeneration or lysosomal storage diseases [3]. Recent reports suggest that autophagy plays a role in the initiation and progression of breast cancer [4], though the underlying molecular mechanisms remain largely unclear.

Autophagosome-lysosome fusion is regulated by many factors, such as Rab7, the HOPS complex, and a set of SNARE proteins [5–7]. Rab7, a small GTPase, is required for sorting acidic hydrolases and forming functional lysosomes [8, 9]. While Rab7 is known to regulate the final step of autophagosome-lysosome fusion [10], only a few Rab7 effector proteins have been characterized in the context of autophagy [11–13]. The effector proteins that co-regulate lysosome acidification and autophagosome-lysosome fusion remain to be explored.

Vacuolar H^+^-ATPases (V-ATPases) are ATP hydrolysis-driven proton pumps essential for membrane trafficking, endocytosis, protein degradation, and the coupled transport of small molecules by regulating the acidification of intracellular vesicles, organelles, and the extracellular milieu in eukaryotes [14, 15]. Notably, V-ATPases have been reported to promote autophagy by regulating lysosomal acidification and autophagosome-lysosome fusion [16, 17]. V-ATPase is a multisubunit complex that includes a membrane-integral V0 domain and a cytosolic V1 domain. The V1 domain consists of eight subunits (A, B, C, D, E, F, G, and H) that hydrolyze ATP, while the V0 domain consists of five subunits (a, c, c″, d, and e) that transport protons. In mammalian cells, V-ATPase also includes two auxiliary subunits (ATP6AP1 and ATP6AP2) [14]. Dysregulation of V-ATPases has been linked to cancer development, metastasis and drug resistance [18]. In breast cancer, V-ATPase inhibition reduces cell invasion and migration, and altered expression of V-ATPase subunits (such as a3 and V1G1) enhances cell invasion and migration [19, 20]. However, the roles of other subunits of V-ATPases in breast cancer remain unclear.

ATP6AP1, an accessory subunit of V-ATPase, is a transmembrane protein that plays a vital role in the assembly and regulation of V-ATPases [21]. ATP6AP1 knockout results in early embryonic lethality, indicating that ATP6AP1 is essential for embryonic development [22]. Moreover, ATP6AP1 regulates a variety of key cellular processes, including membrane trafficking, endocytosis and Ca^2+^-dependent membrane fusion [23, 24]. Despite these functions, the role of ATP6AP1 in autophagy remains unexplored. Recent studies have demonstrated that inactivating mutations of ATP6AP1 drive the tumorigenesis of granular cell tumors (GCTs) [25]. While ATP6AP1 has been identified as a biomarker in breast cancer [26–28], its function in breast cancer is still not well understood.

Here, we found that ATP6AP1 overexpression in luminal breast cancer is associated with poor patient outcomes. We further demonstrated that ATP6AP1 promotes the proliferation of human luminal breast cancer cells and enhances tamoxifen (TAM) resistance by activating autophagy. Mechanistically, our findings show that ATP6AP1 promotes autophagy by regulating V-ATPase-mediated acidification of lysosomes and promoting the interaction between Rab7 and the HOPS complex.

## Results

### ATP6AP1 overexpression in luminal breast cancer is associated with poor patient outcome

**Fig. 1:**
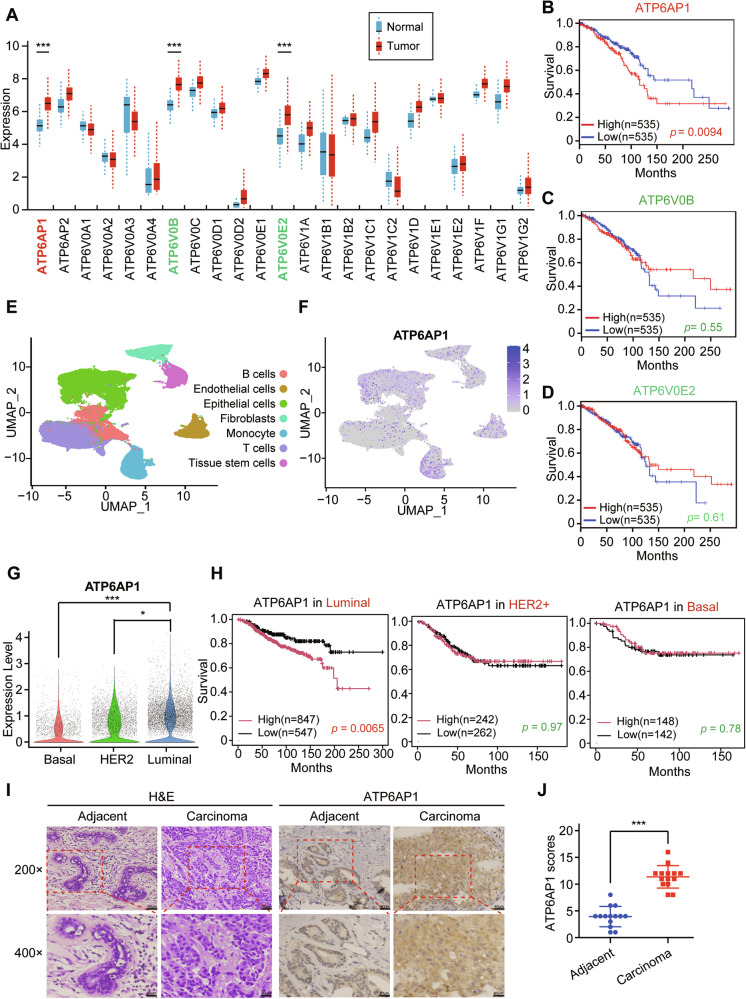
ATP6AP1 is overexpressed in luminal breast cancer cells and correlated with poor luminal breast cancer patient survival. **A** Expression levels of different subunits of V-ATPase in BRCA from the TCGA database were determined by GEPIA. ***p < 0.001. Kaplan‒Meier curves were used to evaluate differences between breast cancer patients with high and low expression of ATP6AP1 **B**, ATP6V0B **C**, and ATP6V0E2 **D** in terms of overall survival. **E** Cell type clusters of eligible single cells based on the expression of canonical marker genes. Feature (UMAP) **F** and Violin **G** plot showing the expression of ATP6AP1 in single cells. ***p < 0.001, *p < 0.05. **H** Kaplan‒Meier survival curve analysis of the prognostic significance of high and low expression of ATP6AP1 in different subclasses of breast cancer using the Kaplan‒Meier Plotter database. **I** HE staining and immunohistochemical detection of ATP6AP1 expression in luminal breast cancer samples and matched adjacent breast tissue samples. Scale bars, 40 μm (above), 20 μm (below). **J** ATP6AP1 expression was plotted based on the immunohistochemical score. Statistical significance was analyzed with the Wilcoxon matched-pairs signed-rank test. *n* = 14, ***p < 0.001.

To explore the function of V-ATPases in breast cancer (BRCA), we first analyzed the expression levels of different subunits of V-ATPase in breast cancer using the Gene Expression Profiling Interactive Analysis (GEPIA) database. The results showed that only three subunits (ATP6AP1, ATP6V0B and ATP6V0E2) were significantly overexpressed in BRCA (Fig. 1A). To assess the clinical relevance of this overexpression, we evaluated overall survival (OS) data from the GEPIA database and found that only ATP6AP1 overexpression was associated with poor overall survival (Fig. 1B–D). Thus, we selected the auxiliary subunit ATP6AP1 as a candidate gene for further investigations.

Human breast cancer consists of at least four molecularly distinct subtypes defined as: luminal A, luminal B, basal-like, and human epidermal growth factor receptor 2 (HER2)-enriched subtypes. Luminal breast cancers, which include the Luminal A and Luminal B subtypes, express estrogen receptor α (ERα), whereas the other subtypes contain ERα-negative tumors[29]. Using single-cell sequencing data (GSE176078) [30], we found that ATP6AP1 was predominantly expressed in epithelial cells and monocytes (Fig. 1E, F). Therefore, we applied inferCNV analysis to epithelial cells to identify malignant cells, with T cells and endothelial cells as negative controls. In addition, PAM50 was used to classify malignant epithelial cells. The expression of ATP6AP1 in breast cancer subtypes obtained from scRNA-seq showed that the luminal subtype of breast cancer had the highest ATP6AP1 transcript levels among all subtypes (Fig. 1G). Moreover, survival analysis subtypes from the Kaplan‒Meier Plotter database [31] showed that high ATP6AP1 expression was associated with poor patient outcomes in luminal breast cancer, while it was insignificant in HER2+ or basal (Fig. 1H), consistent with findings from the bc-GenExMiner tool (Fig. S1A). Furthermore, we assessed ATP6AP1 protein levels in primary human luminal breast cancer samples and matched adjacent breast tissue samples using immunohistochemistry (IHC). The results demonstrated significantly higher ATP6AP1 protein expression in luminal breast cancer tissues compared to adjacent breast tissues (Fig. 1I, J). In addition, consistent results were obtained through Western blot analysis for the detection of ATP6AP1 protein expression (Fig. S1B, C). These findings suggest that ATP6AP1 is overexpressed in luminal breast cancer and may serve as an independent predictive and prognostic biomarker for luminal breast cancer.

### ATP6AP1 promotes human luminal breast cancer cell proliferation and TAM resistance

**Fig. 2:**
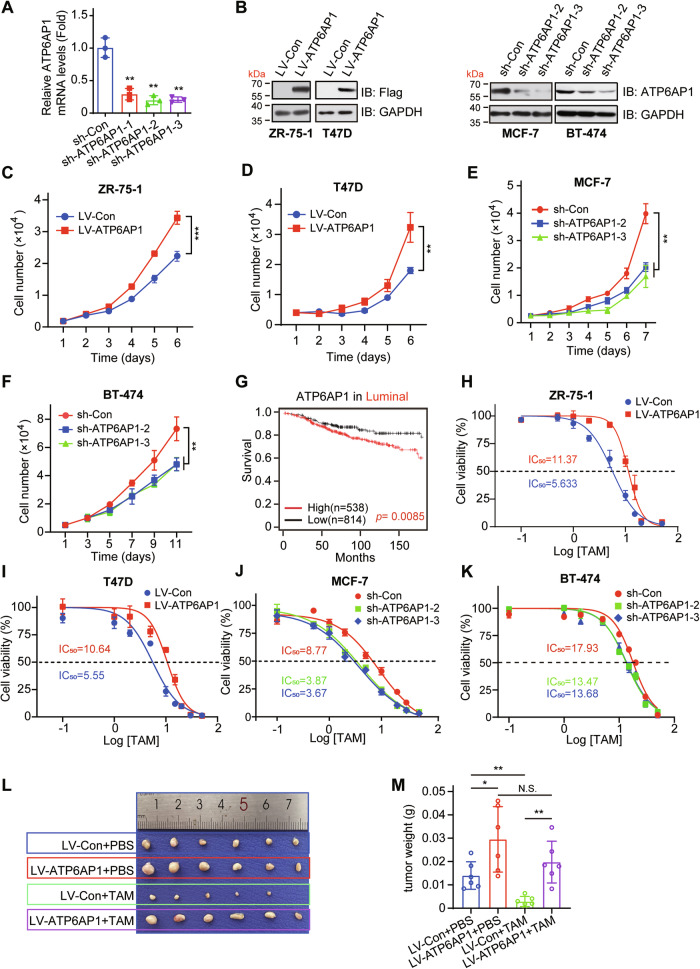
ATP6AP1 promotes human luminal breast cancer cell growth and TAM resistance. **A** The knockdown efficiency of shRNAs against ATP6AP1 in MCF-7 cells was detected using RT-qPCR. The bar graph displays the mean ± SD, *n* = 3, **p < 0.01. **B** ATP6AP1 was stably overexpressed using lentivirus in ZR-75-1 and T47D cells, and stably knocked down using lentivirus in MCF-7 and BT-474 cells. The ATP6AP1 protein expression level was detected by Western blotting. **C, D** ATP6AP1 overexpression promotes cell proliferation in ZR-75-1 and T47D cells. Cell proliferation was determined by flow cytometry on the indicated days. The graph displays the mean ± SD, *n* = 3, **p < 0.01, ***p < 0.001. **E, F** ATP6AP1 knockdown with specific shRNA inhibits cell proliferation in MCF-7 and BT-474 cells. Cell proliferation was determined by flow cytometry on the indicated days. The graph displays the mean ± SD, *n* = 3, **p < 0.01. **G** Kaplan‒Meier survival curve analysis of the prognostic significance of high and low ATP6AP1 expression in luminal breast cancer patients receiving tamoxifen chemotherapy using the Kaplan‒Meier plotter database. Cells were treated with concentration-gradient TAM for 72 hours, and then TAM resistance was verified using CCK-8 assay. ATP6AP1 overexpression promotes TAM resistance in ZR-75-1 cells **H** and T47D cells **I**, whereas ATP6AP1 knockdown enhances TAM sensitivity in MCF-7 cells **J** and BT-474 cells **K**. *n* = 3. **L** Xenograft mouse models using ZR-75-1 cells stably overexpressing ATP6AP1 or empty vector. ATP6AP1 overexpression increased the tumor volume and weight, and ATP6AP1 overexpression reduced sensitivity to TAM-mediated tumor growth inhibition. **M** Quantification of tumor weight in **L**. The bar graph displays the mean ± SD, *n* = 6, *p < 0.05, **p < 0.01, N.S., no significance.

In order to elucidate the role of ATP6AP1 in luminal breast cancer, we selected luminal breast cancer cell lines for subsequent studies, including ER-positive, PR-positive luminal A cells MCF-7, T47D, ZR-75-1, and ER-positive, HER2-positive luminal B cells BT-474. To investigate the role of ATP6AP1 in luminal breast cancer cell proliferation, we generated cell lines with ATP6AP1 overexpression (ZR-75-1 and T47D cells) or knocking down (MCF-7 and BT-474 cells) using lentivirus. These manipulations were confirmed by RT‒qPCR (Fig. 2A) and Western blotting (Fig. 2B). ATP6AP1 overexpression enhanced cell proliferation (Fig. 2C, D), whereas ATP6AP1 depletion reduced cell proliferation (Fig. 2E, F), indicating that ATP6AP1 promotes luminal breast cancer cell proliferation in vitro.

Endocrine therapies such as tamoxifen are classical treatments for ERα-positive breast cancer, especially for Luminal A subtype [32], therefore, we verified whether ATP6AP1 would have an effect on the drug sensitivity of TAM-treated luminal breast cancer cells. To assess whether ATP6AP1 affects TAM resistance, we first analyzed the prognostic significance of the altered expression of ATP6AP1 in luminal breast cancer patients who received TAM chemotherapy using the Kaplan‒Meier plotter database. Patients with higher ATP6AP1 levels had much lower OS rates following TAM treatment (Fig. 2G). Next, to evaluate the role of ATP6AP1 in TAM sensitivity, the half-maximal inhibitory concentration (IC_50_) of TAM was measured using a CCK-8 assay. We found that the overexpression of ATP6AP1 enhanced TAM resistance in ZR-75-1 and T47D cells (Fig. 2H, I), whereas the depletion of ATP6AP1 reduced TAM resistance in MCF-7 and BT-474 cells (Fig. 2J, K). These results suggest that ATP6AP1 promotes TAM resistance in luminal breast cancer cells. To further validate the role of ATP6AP1 in cell proliferation and TAM sensitivity, we established breast cancer xenograft mouse models using ZR-75-1 cells stably overexpressing ATP6AP1 or a control vector. Compared to the control group, ATP6AP1 overexpression drastically increased the tumor volume and weight, moreover, ATP6AP1 overexpression reduced sensitivity to TAM-mediated tumor growth inhibition (Fig. 2L, M). We verified ATP6AP1 expression in xenograft tumors by immunohistochemistry and western blotting experiments (Fig. S2A, B). These findings demonstrate that ATP6AP1 promotes luminal breast cancer cell proliferation and TAM resistance.

### ATP6AP1 overexpression promotes autophagy in luminal breast cancer cells

**Fig. 3:**
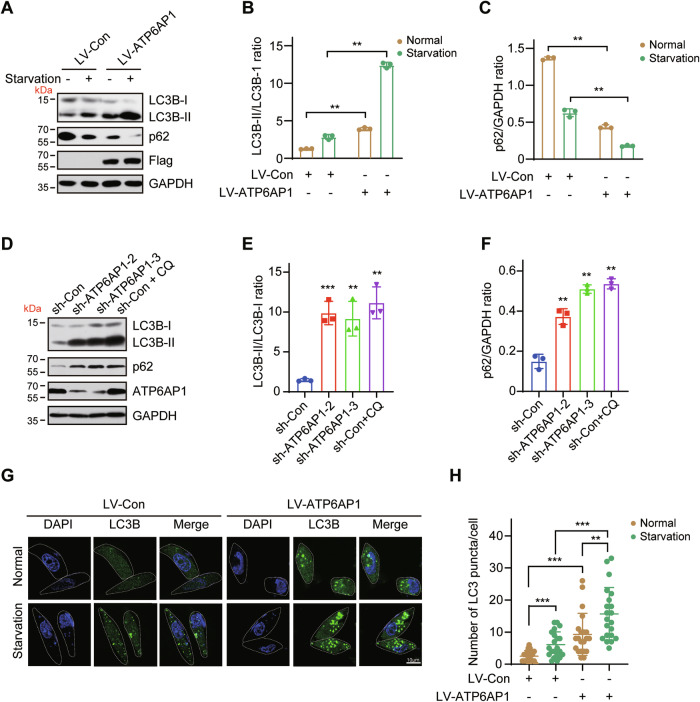
ATP6AP1 overexpression promotes autophagy. **A** ATP6AP1 overexpression induces autophagy in ZR-75-1 cells. ZR-75-1 cells were infected with LV-Con or LV-ATP6AP1 to establish stable cell lines. Cells were cultured in complete RPMI 1640 medium or EBSS for 4 h, and Western blotting was performed. Quantification of **A** using ImageJ software. Summarized data showing densitometric measurements of LC3B-II: LC3B-I **B** and p62: GAPDH **C**, GAPDH as loading control. The bar graph displays the mean ± SD, *n* = 3, **p < 0.01. **D** ATP6AP1 knockdown inhibits autophagy in MCF-7 cells. MCF-7 cells were infected with LV-sh-Con or LV-sh-ATP6AP1 for 72 h, and Western blotting was performed. CQ was used as a positive control for autophagy inhibition. Quantification of **D** using ImageJ software. Summarized data showing densitometric measurements of LC3B-II: LC3B-I **E** and p62: GAPDH **F**. GAPDH was used as the loading control. The bar graph displays the mean ± SD, *n* = 3, **p < 0.01, ***p < 0.001. **G** ATP6AP1 overexpression increases LC3 puncta. ZR-75-1 cells were infected with LV-Con or LV-ATP6AP1 and cultured in complete RPMI 1640 medium or EBSS for 4 h. Cells were fixed and stained with anti-LC3B antibody and DAPI. The distribution of LC3B protein was visualized with a fluorescence microscope. Scale bars, 10 μm. **H** Quantification of LC3-positive puncta per cell. Mean ± SD, **p < 0.01, ***p < 0.001 by two-way ANOVA (*n* = 20).

Previous studies have shown that ATP6AP1, an accessory subunit of V-ATPase, plays a crucial role in the assembly and regulation of V-ATPase [21]. To determine the role of ATP6AP1 in autophagy, we examined its effects using ZR-75-1 cells stably overexpressing ATP6AP1 and MCF-7 cells stably knocking down ATP6AP1. We assessed the autophagy marker LC3B and autophagy substrate p62/SQSTM1 through Western blotting. Overexpression of ATP6AP1 induced the turnover of LC3B-I into LC3B-II and decreased the protein level of p62 under both normal and starvation conditions (Fig. 3A–C), indicating that ATP6AP1 activates autophagy. LC3-II, is commonly used to monitor the amount of autophagosome formation in cells [33], and an increase in LC3-II can reflect either induction of autophagy or a blockage in later stages of the autophagy pathway, such as autophagosome-lysosome fusion and/or lysosomal degradation [34]. Chloroquine (CQ) is often used as a lysosomal inhibitor to measure autophagic flux. It was shown that CQ treatment resulted in significantly higher levels of LC3-II than control cells, which may impair basal autophagic flux by reducing autophagosome-lysosome fusion, leading to substantial accumulation of LC3-II [35]. Similarly, depletion of ATP6AP1 caused a dramatic accumulation of LC3B-II and p62, consistent with the effect of CQ treatment, indicating a block in autophagic flux (Fig. 3D–F). During the formation of autophagosomes, LC3 relocates from the cytoplasm, in which it has a diffuse distribution, to autophagosomes; the punctate distribution of LC3 corresponds to that of autophagosomes. As expected, ATP6AP1 overexpression significantly increased LC3 puncta formation under both normal and starvation conditions compared to control cells (Fig. 3G, H). Taken together, these results indicate that ATP6AP1 promotes autophagy.

### ATP6AP1 regulates lysosome acidification

**Fig. 4:**
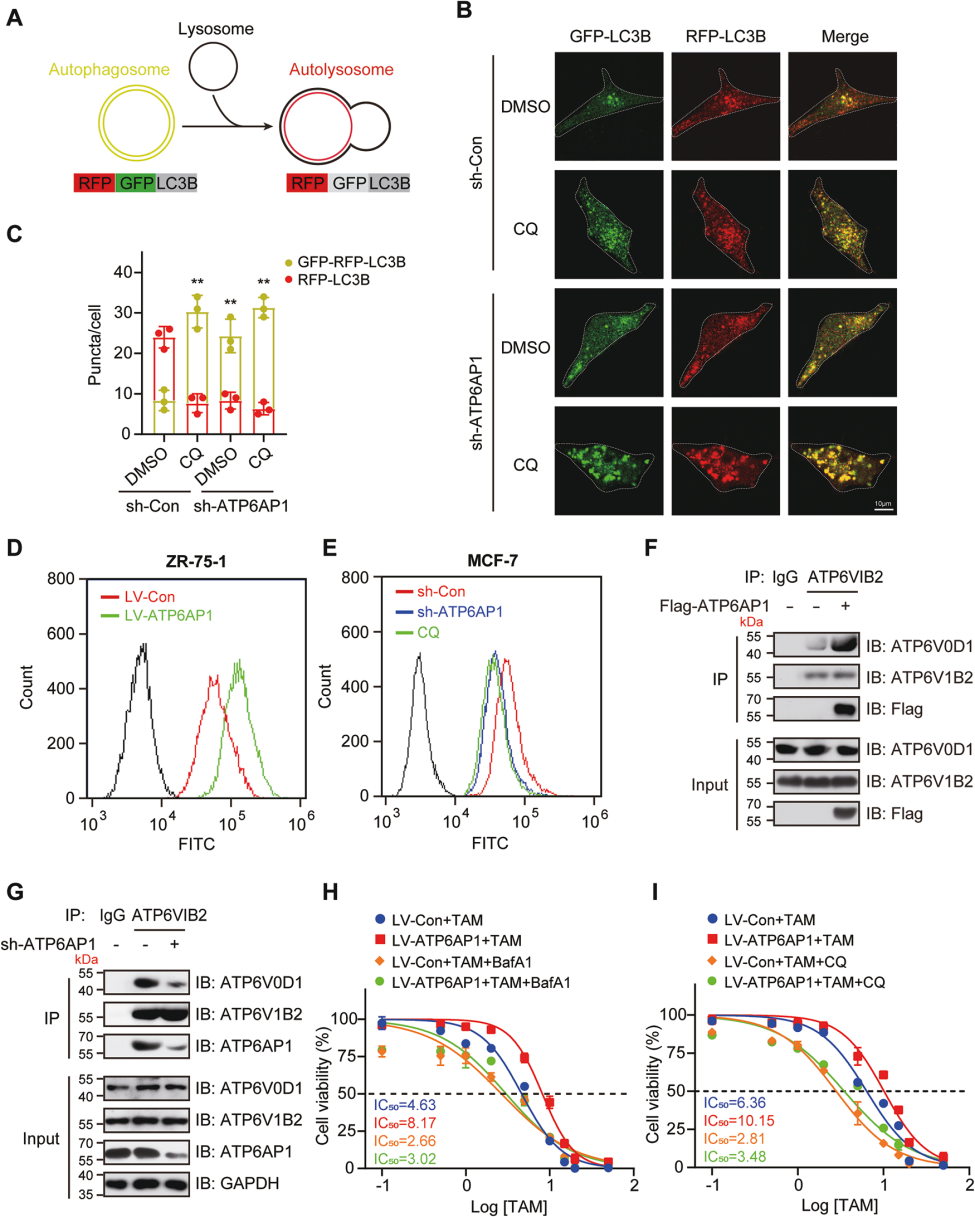
ATP6AP1 promotes autophagy by regulating V-ATPase-mediated acidification of lysosomes. **A** The autophagy tandem sensor RFP-GFP-LC3B was used to measure autophagic flux. GFP is sensitive to acidic pH, and when autophagosomes fuse with lysosomes to form autolysosomes, GFP signals are quenched at lysosomal pH. Hence, autophagosomes (GFP + RFP + , yellow puncta) and autolysosomes (GFP- RFP + , red puncta) were observed. **B** ATP6AP1 knockdown inhibits autolysosome formation. MCF-7 cells were infected with LV-sh-Con or LV-sh-ATP6AP1 for 72 h and then treated with vehicle DMSO or chloroquine (10 μM) for 12 h. Green and red fluorescence was detected by confocal microscopy. Scale bars, 10 μm. **C** Quantification of autophagic flux as analyzed in **B**. The average percentage of yellow puncta and red puncta per cell was calculated. The bar graph displays the mean ± SD, *n* = 3, **p < 0.01. **D, E** ATP6AP1 promotes lysosomal acidification. ZR-75-1 cells infected with LV-Con or LV-ATP6AP1 and MCF-7 cells infected with LV-sh-Con or LV-sh-ATP6AP1 were stained with 5 mM LysoSensor Green for 2 h, and green fluorescence was detected by flow cytometry. CQ, used as a positive control, increased the lysosomal pH. **F** ATP6AP1 promotes the assembly of V-ATPase. Ectopic ATP6AP1 increases ATP6V0D1-ATP6V1B2 interactions. HEK-293T cells transfected with Flag-ATP6AP1 plasmids were subjected to a co-IP assay. **G** ATP6AP1 knockdown decreases ATP6V0D1-ATP6V1B2 interactions. MCF-7 cells infected with LV-sh-Con or LV-sh-ATP6AP1 were subjected to a co-IP assay. Overexpression of ATP6AP1 promotes TAM resistance in T47D cells. However, TAM resistance was inhibited by adding 10 μm BafA1 **H** or 20 μm CQ **I** for 72 h, *n* = 3.

Lysosomal acidification and autophagosome-lysosome fusion are essential for lysosomal degradation, the late steps in the autophagic process, and the maintenance of functional autophagic flux [17]. Given that V-ATPase is a universal proton pump and ATP6AP1 is an important accessory subunit for V-ATPase assembly [21], we hypothesized that ATP6AP1 promotes autophagy by regulating the acidification of lysosomes. To test this hypothesis, we used the autophagy tandem sensor RFP-GFP-LC3B to monitor autophagic flux. RFP is an acid-stable protein that retains fluorescence when autophagosomes fuse with lysosomes, while GFP fluorescence is lost in lysosomes (Fig. 4A). MCF-7 cells were co-infected with LV-sh-ATP6AP1 and LV-mCherry-GFP-LC3, and puncta formation was monitored. As CQ blocked autophagic flux, depletion of ATP6AP1 induced a significant increase in the number of nonacidic autophagosomes and a large decrease in the number of acidic autolysosomes (Fig. 4B, C), suggesting that loss of ATP6AP1 disrupts autophagic flux. Similarly, we co-infected T47D cells with LV-ATP6AP1 and LV-mCherry-GFP-LC3 and monitored autophagic flux. The overexpression of ATP6AP1 induced a significant increase in the number of acidic autophagosomes regardless of starvation conditions (Fig. S3A, B), suggesting that ATP6AP1 promotes autophagic flux. Moreover, we found that overexpression of ATP6AP1 enhanced lysosomal acidification (Fig. 4D), whereas depletion of ATP6AP1 blocked lysosomal acidification (Fig. 4E). We also investigated ATP6AP1’s role in V-ATPase assembly in lysosomes. The assembly of V-ATPase was evaluated by the interaction between ATP6V1B2 (V1 subunit) and ATP6V0D1 (V0 subunit). As expected, overexpression of ATP6AP1 enhanced the interaction between ATP6V1B2 and ATP6V0D1 (Fig. 4F), whereas depletion of ATP6AP1 significantly reduced this interaction (Fig. 4G), suggesting ATP6AP1 facilitates the assembly of V-ATPase in lysosomes. To further explore the impact of ATP6AP1 on TAM resistance, we verified the effect of CQ (an autophagy inhibitor) or Bafilomycin-A1 (BafA1, a V-ATPase inhibitor) on TAM resistance in luminal breast cancer cells. Overexpression of ATP6AP1 enhanced TAM resistance in T47D cells, however, this effect was abolished by treatment with CQ or BafA1, resulting in significantly reduced TAM resistance (Fig. 4H, I). These findings suggest that ATP6AP1 promotes autophagy and TAM resistance by regulating V-ATPase-mediated lysosomal acidification.

### ATP6AP1 regulates autophagosome-lysosome fusion

**Fig. 5:**
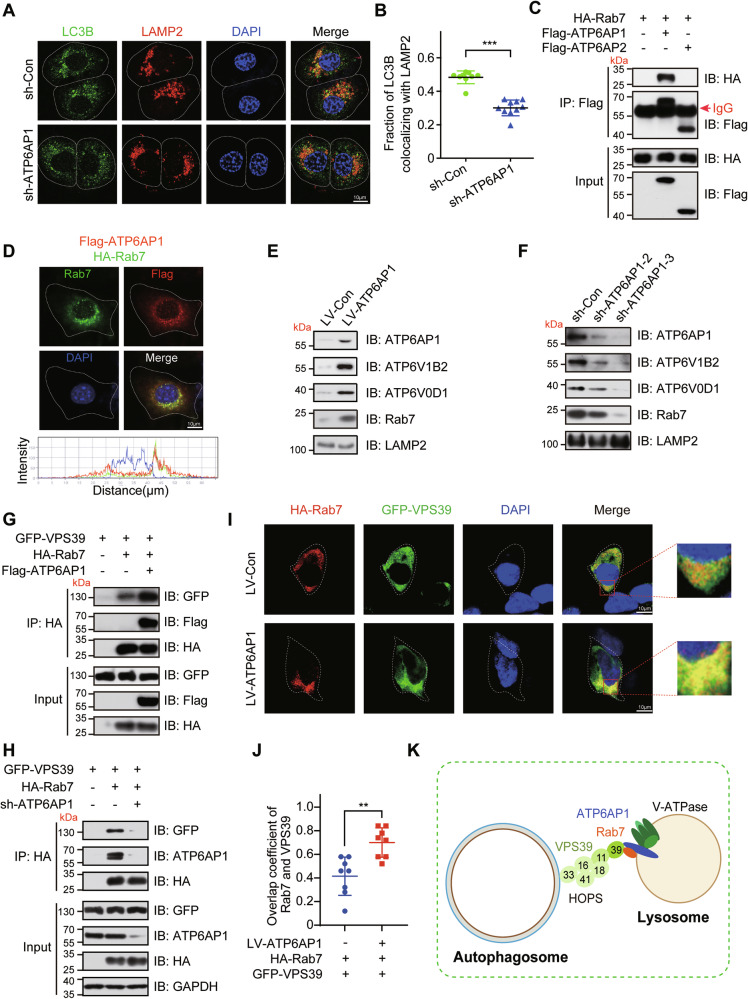
ATP6AP1 promotes autophagosome-lysosome fusion by regulating the interaction between Rab7 and the HOPS complex. **A** Knockdown of ATP6AP1 reduces co-localization of LC3B with LAMP2. Representative immunofluorescence images of the colocalization of LC3B and LAMP2 in MCF-7 cells infected with LV-sh-Con or LV-sh-ATP6AP1 were shown. Scale bars, 10 μm. **B** Quantification of LC3B-LAMP2 colocalized voxels (volumetric pixels). Mean ± SD, ***p < 0.001 by two-way ANOVA (*n* = 10). **C** ATP6AP1 interacts with Rab7. HA-Rab7 interacts with Flag-ATP6AP1 but not Flag-ATP6AP2. HEK-293T cells transfected with HA-Rab7 and Flag-ATP6AP1 or Flag-ATP6AP2 plasmids for 48 h were subjected to a co-IP assay. **D** ATP6AP1 co-localizes with Rab7 in T47D cells. Representative immunofluorescence images showing the colocalization of Rab7 and ATP6AP1 in T47D cells infected with Flag-ATP6AP1 and HA-Rab7. Scale bars, 10 μm. **E** ATP6AP1 increases the protein level of Rab7 in lysosomes. Lysosomal proteins were extracted from 2 × 10^7^ T47D cells, and Western blotting analysis was performed to determine protein levels of Rab7, ATP6V0D1, ATP6V1B2, ATP6AP1, and LAMP2. **F** Knockdown of ATP6AP1 reduced the protein level of Rab7 in lysosomes. Lysosomal proteins were extracted from 2 × 10^7^ MCF-7 cells, and Western blotting analysis was performed to determine protein levels of Rab7, ATP6V0D1, ATP6V1B2, ATP6AP1, and LAMP2. **G** ATP6AP1 increases Rab7-VPS39 interactions. HEK-293T cells co-transfected with HA-Rab7 and GFP-VPS39 with or without Flag-ATP6AP1 plasmids for 48 h were subjected to a co-IP assay. **H** ATP6AP1 knockdown decreases Rab7-VPS39 interactions. HEK-293T cells co-transfected with HA-Rab7 and GFP-VPS39 with or without sh-ATP6AP1 plasmids for 48 h were subjected to a co-IP assay. **I** ATP6AP1 increases co-localization of Rab7 with VPS39. Representative immunofluorescence images showing the colocalization of Rab7 and VPS39 in T47D cells infected with Flag-ATP6AP1 and transfected with HA-Rab7 and GFP-VPS39. Scale bars, 10 μm. **J** Quantification of Rab7-VPS39 colocalized voxels (volumetric pixels). Mean ± SD, ***p < 0.001 by two-way ANOVA (*n* = 8). **K** The Schematic shows how ATP6AP1 promotes autophagosome-lysosome fusion by regulating the interaction between Rab7 and the HOPS complex.

Next, we evaluated whether ATP6AP1 regulates autophagosome-lysosome fusion by examining the colocalization of LC3B with LAMP2. We observed a decrease in the percentage of LC3B-positive autophagosomes that colocalized with LAMP2-positive lysosomes in ATP6AP1-depleted cells (Fig. 5A, B), suggesting that depletion of ATP6AP1 impairs autophagosome-lysosome fusion. To further elucidate how ATP6AP1 regulates autophagosome-lysosome fusion, we investigated its interaction with known regulators of autophagosome-lysosome fusion. Notably, our protein-protein interaction (PPI) network analysis indicated a potential interaction between ATP6AP1 and Rab7. This interaction was confirmed by a co-immunoprecipitation (Co-IP) assay, which showed that Rab7 associates with ATP6AP1 but not with ATP6AP2, another accessory V-ATPase subunit (Fig. 5C). We also confirmed the colocalization of ATP6AP1 and Rab7 in the cytoplasm by immunofluorescence assays (Fig. 5D). To verify the effect of ATP6AP1 on Rab7, we isolated lysosomes from cells and examined the protein level of Rab7 in lysosomes. Interestingly, we found that overexpression of ATP6AP1 increased the protein levels of Rab7, ATP6V1B2, and ATP6V0D1 in lysosomes of T47D cells, with LAMP2 serving as a positive control (Fig. 5E). Conversely, knockdown of ATP6AP1 in MCF-7 cells decreased the protein level of Rab7 in lysosomes, as well as ATP6V1B2 and ATP6V0D1 (Fig. 5F). These results suggest that ATP6AP1 recruits Rab7 to lysosomes through interaction with the Rab7.

Rab7 plays an important role in autophagosome-lysosome fusion [36, 37]. Rab7 localizes to lysosomes and promotes the fusion of autophagosomes with lysosomes by working with HOPS effectors and specific members of the SNARE family [7, 38]. However, the mechanism regulating the interaction between Rab7 and the HOPS complex remains unclear. Given ATP6AP1’s role as a transmembrane protein localized to the lysosome, we hypothesized that ATP6AP1 might modulate the interaction between Rab7 and the HOPS complex. To investigate this conjecture, we altered ATP6AP1 expression to monitor its effects on the interaction between Rab7 and VPS39 (a subunit of the HOPS complex). We found that overexpression of ATP6AP1 enhanced the interaction between Rab7 and VPS39, whereas depletion of ATP6AP1 significantly reduced this interaction (Fig. 5G, H). Furthermore, we verified that ATP6AP1 overexpression promoted Rab7 and VPS39 colocalization in the cytoplasm (Fig. 5I, J), whereas ATP6AP1 depletion inhibited the co-localization of Rab7 and VPS39 in the cytoplasm (Fig. S4A, B), indicating that ATP6AP1 facilitates autophagosome-lysosome fusion by enhancing the interaction between Rab7 and the HOPS complex. Taken together, our findings revealed that ATP6AP1 activates autophagy by facilitating both lysosome acidification and autophagosome-lysosome fusion (Fig. 5K).

### ATP6AP1 promotes human luminal breast cancer cell proliferation and TAM resistance by activating autophagy

**Fig. 6:**
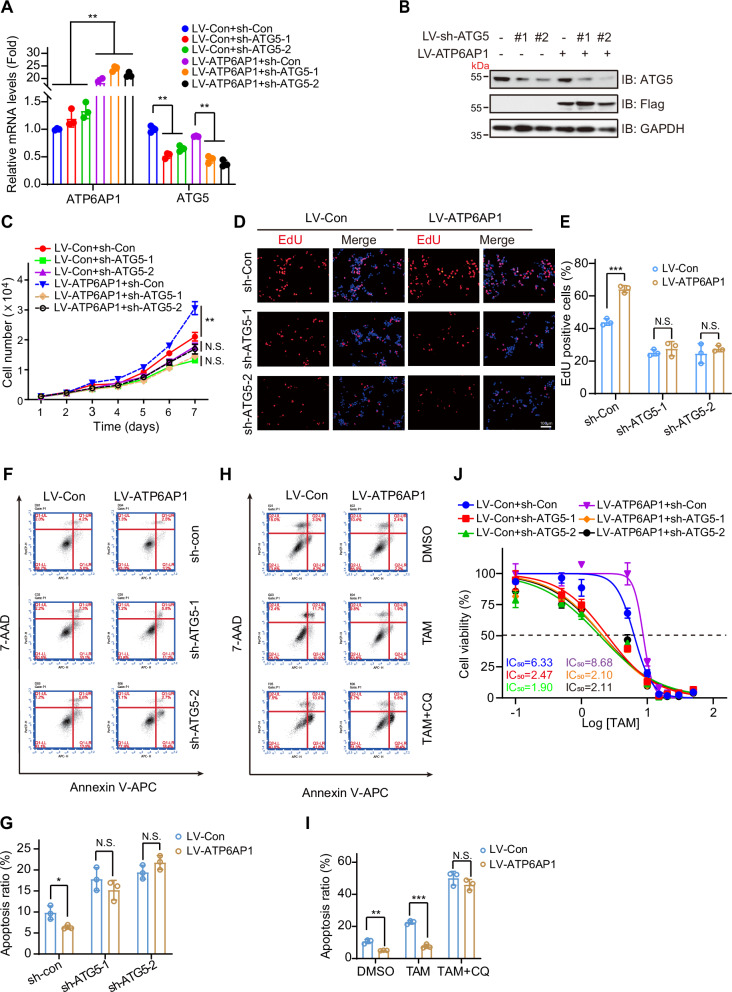
ATP6AP1 promotes luminal breast cancer cell growth and TAM resistance by regulating autophagy. **A** ZR-75-1 cells were infected with ATP6AP1 overexpression (LV-ATP6AP1) and ATG5 knockdown (LV-sh-ATG5) lentiviruses and harvested for RT‒qPCR. The bar graph displays the mean ± SD, *n* = 3, **p < 0.01. **B** ZR-75-1 cells were infected with ATP6AP1 overexpression (LV-ATP6AP1) and ATG5 knockdown (LV-sh-ATG5) lentiviruses and harvested for western blotting. **C** ATP6AP1 facilitates luminal breast cancer cell proliferation by activating autophagy. ZR-75-1 cells were infected with ATP6AP1 overexpression (LV-ATP6AP1) and ATG5 knockdown (LV-sh-ATG5) lentiviruses. Cell proliferation was determined by flow cytometry. The graph displays the mean ± SD, *n* = 3, **p < 0.01, N.S., no significance. **D** Cell proliferation of ZR-75-1 cells was determined by EdU assay. Scale bars, 100 μm. **E** Quantification of the data in **D**. The bar graph displays the mean ± SD, *n* = 3, ***p < 0.001, N.S., no significance. **F** Knockdown of ATG5 blocked ATP6AP1-attenuated apoptosis. T47D cells infected with LV-ATP6AP1 or LV-sh-ATG5 were treated with EBSS for 4 h and stained with annexin V and 7-AAD to determine the apoptosis rate. **G** Quantification of the data in **F**. The bar graph displays the mean ± SD, *n* = 3, *p < 0.05, N.S., no significance. **H** CQ attenuated ATP6AP1-induced TAM resistance. ZR-75-1 cells were infected with LV-ATP6AP1 or empty vector, treated with DMSO, TAM + PBS, or TAM + CQ for 72 h, and then stained with annexin V and 7-AAD to determine the apoptosis rate. **I** Quantification of the data in **H**. The bar graph displays the mean ± SD, *n* = 3, **p < 0.01, ***p < 0.001, N.S., no significance. **J** Overexpression of ATP6AP1 promotes TAM resistance in T47D cells. However, ATG5 depletion inhibited this effect. Cells were treated with concentration-gradient TAM for 72 h, and then TAM resistance was verified using CCK-8 assay, *n* = 3.

ATG proteins play a central role in the autophagy process by regulating the initiation and maturation of autophagosomes [39]. Among them, *Atg5* is an important gene in mammalian autophagy, serving as a key promoter of early autophagy [40].To determine whether ATP6AP1 promotes luminal breast cancer cell proliferation and TAM resistance through autophagy activation, we blocked autophagy by knocking down ATG5 using specific shRNA (Figs. 6A, B, S5A, B). We observed that the knockdown of ATG5 significantly reduced the formation of LC3 puncta induced by ATP6AP1 overexpression (Fig. S5C, D). Moreover, ATG5 knockdown effectively prevented the increase in luminal breast cancer cell proliferation caused by ATP6AP1 overexpression (Figs. 6C–E, S5E–G). In addition, we induced apoptosis by starvation treatment of breast cancer cells. ATP6AP1 overexpression reduced starvation-induced apoptosis compared to controls, and this protective effect was abolished by ATG5 knockdown (Fig. 6F, G), suggesting that ATG5 is necessary for ATP6AP1-induced autophagy.

Furthermore, we used CQ to inhibit autophagy in ATP6AP1-overexpressing breast cancer cells and analyzed the impact on TAM-induced apoptosis. Compared to the control group, ATP6AP1 overexpression decreased TAM-induced apoptosis, which was abrogated by CQ treatment (Fig. 6H, I), suggesting that inhibiting autophagy attenuated ATP6AP1-induced TAM resistance. TAM resistance experiments also confirmed that depleting ATG5 increased TAM sensitivity in T47D cells and blocked ATP6AP1-induced TAM resistance (Fig. 6J). Combined, these data indicate that ATP6AP1 overexpression promotes luminal breast cancer cell proliferation and TAM resistance through autophagy activation.

## Discussion

V-ATPase is a multi-subunit complex essential for establishing and maintaining pH homeostasis in endosomes and lysosomes via ATP hydrolysis-driven transport of protons [14, 15]. Recent studies have shown that abnormal expression of specific subunits of V-ATPase correlates with cancer progression [20]. Here, we reported that aberrant overexpression of ATP6AP1 promoted cell proliferation and TAM resistance in luminal breast cancer (Fig. 2). Previous research identified ATP6AP1 as a breast cancer-specific biomarker for early detection [26, 27] and was recently found to be a potential prognostic biomarker [28]. Consistent with this, we found that ATP6AP1 overexpression in luminal breast cancer was associated with poor patient outcomes (Fig. 1). Therefore, ATP6AP1 could serve as both a specific biomarker and a potential therapeutic target for luminal breast cancer.

Breast cancer is one of the most common cancers in the world, with high morbidity and mortality. It consists of at least four molecularly distinct subtypes defined as: luminal A, luminal B, basal-like, and human epidermal growth factor receptor 2 (HER2)-enriched subtypes. Luminal breast cancers express estrogen receptor α (ERα), whereas the other subtypes contain ERα-negative tumors [29]. The luminal A tumors are characterized by the high expression of estrogen receptor and progesterone receptor (PgR)-related genes and low expression of proliferation-related genes [41], whereas luminal B tumors are characterized by the low expression of estrogen receptor and progesterone receptor genes [42–44], and the high expression of proliferation cluster genes (MKI67) [45]. Approximately 20% of luminal B tumors show HER2 positivity [46]. Hormonal therapy is the cornerstone of treatment for luminal breast cancer, with selective estrogen receptor modulators (SERMs) such as tamoxifen (TAM) being the first line of defense. However, the development of resistance to TAM is a significant clinical challenge. We discovered that ATP6AP1 enhances the TAM resistance of luminal breast cancer cells by activating autophagy. This study provides new insights into TAM resistance in breast cancer.

The dual nature of autophagy in tumors has garnered considerable attention in the field of cancer research. Autophagy, a highly conserved cellular process, plays a complex and often paradoxical role in tumor progression. On one hand, autophagy can act as a tumor suppressor by maintaining genomic stability, eliminating damaged organelles, and preventing the accumulation of toxic protein aggregates, which helps to maintain cellular homeostasis and prevent the initiation of tumorigenesis [47]. For example, in certain contexts, the induction of autophagy can lead to cell cycle arrest and promote cell death, thereby inhibiting tumor growth [47–49]. On the other hand, autophagy can also promote tumor progression once a tumor has been established. It can provide cancer cells with nutrients and energy under conditions of metabolic stress, such as hypoxia and nutrient deprivation, which are common in the tumor microenvironment. This adaptive response allows cancer cells to survive and continue to proliferate [50, 51]. Moreover, autophagy can contribute to the resistance of cancer cells to various therapeutic agents, including chemotherapy and radiation therapy, by reducing the levels of reactive oxygen species (ROS) and preventing the activation of cell death pathways [52, 53]. While targeting autophagy emerges as a tantalizing therapeutic avenue, it necessitates a meticulous appraisal of the tumor’s specific context and stage. Understanding the dual roles of autophagy in tumors is crucial for the development of effective cancer therapies.

The role of autophagy in drug resistance during cancer treatments is well documented. TAM is the primary endocrine therapy currently, and it is a principal treatment for luminal breast cancer, yet TAM resistance remains a significant issue [54]. Autophagy is a major cause of tamoxifen resistance in breast cancer [55]. Our study provides proof that ATP6AP1 enhances TAM resistance by activating autophagy, which carries significant implications for the clinical management of luminal breast cancer (Fig. 6). Mechanistically, we found that ATP6AP1 activates autophagy by facilitating lysosome acidification (Fig. 4) and autophagosome-lysosome fusion (Fig. 5). Additionally, we have shown that the blockage of autophagy prevents ATP6AP1-induced TAM resistance, as evidenced by the use of two specific autophagy inhibitors, chloroquine (CQ) and bafilomycin A1 (BafA-1), suggesting that ATP6AP1 promotes TAM resistance through autophagy activation (Fig. 4). Furthermore, we blocked autophagy by knocking down ATG5, and demonstrated that ATP6AP1 stimulates luminal breast cancer cell proliferation and TAM resistance through autophagy activation (Fig. 6). However, substantial progress is required not only in the laboratory but also in the clinic to certify the role of ATP6AP1 as a therapeutic option for breast cancer patients.

The role of ATP6AP1 in cancer is currently not well understood. Fresia Pareja et al. found that loss-of-function mutations in ATP6AP1 likely drive granular cell tumors (GCTs) based on decreased V-ATPase activity and endosomal acidification [25]. In our study, we revealed that ATP6AP1 overexpression promotes luminal breast cancer cell proliferation and TAM resistance by activating autophagy. Mechanistically, we demonstrated that ATP6AP1 induces autophagy by regulating lysosomal acidification as well as autolysosome formation. Thus, ATP6AP1 may influence tumor progression through different mechanisms depending on the cancer type.

Interestingly, we found both ATP6AP1 overexpression and knockdown elevated protein levels of LC3B-II in Fig. 3. Overexpression of ATP6AP1 induced the conversion of LC3B-I to LC3B-II; thus, LC3B-II was elevated while LC3B-I was decreased. When knocking down ATP6AP1, similar to the effect of chloroquine (CQ), blocked the autophagic flux, depletion of ATP6AP1 blocked autophagic flux leading to the accumulation of both LC3B-I and LC3B-II (Fig. 3). It is known that the accumulation of LC3B-II can occur through two primary mechanisms: promotion of autophagosome formation and inhibition of LC3B-II degradation. Recently, several studies have shown that chloroquine inhibits the autophagic flux, causing the accumulation of LC3B-I, LC3B-II, and P62 [56–58]. For example, Cao et al. treated cells with the lysosomal inhibitor CQ and found that LC3B-II accumulation induced by CQ in NSCLC cell lines [59], which aligns with our findings.

The small GTPase Rab7 is an essential organizer of receptor recycling and lysosomal degradation by recruiting a variety of effectors. The effectors of Rab7 play multiple roles in the endolysosome and autophagy‒lysosome pathways, such as RILP (Rab-interacting lysosomal protein), NRBF2 (nuclear receptor binding factor 2), WDR91 (WD repeat domain 91), Nlp (ninein-like protein), FYCO1 (FYVE and coiled-coil containing 1), PI4P (phosphatidylinositol-4-phosphate) and vici syndrome protein EPG5. For instance, Nlp and FYCO1 bind to LC3 and PI3P to mediate end-directed vesicle transport and facilitate the formation of autolysosomes [12, 60]. NRBF2 binds to PtdIns3K to regulate Rab7 activity for autophagy maturation and PI4P controls Rab7 membrane cycling during autophagosome-lysosome fusion [61, 62]. RILP and WDR91 interact with the HOPS complex to facilitate Rab7-dependent lysosome fusion [56, 58]. These Rab7 effectors colocalize with Rab7 on the lysosome membrane but not transmembrane proteins. In our study, we revealed that ATP6AP1 acts as a Rab7 effector protein, playing dual functions in lysosome acidification and autophagosome-lysosome fusion (Fig. 4).

Each subunit of V-ATPase drives a specific mechanism that controls pump activation and regulation. Apart from this, some subunits function in the regulation of diverse biomechanisms. For instance, anterograde trafficking of secretory lysosomes is predominantly orchestrated by V-ATPase’s a3 subunit, which is pivotal in the process of bone resorption by osteoclasts [57]. Recent studies have shown that V-ATPase a1 and a3 subunits differentially regulate phagosome maturation, lysosomal acidification, and autophagy in microglia [63]. ATP6AP2/PRR serves as the (pro) renin receptor, playing significant roles not only in the renin-angiotensin system, which is crucial for blood pressure regulation but also in electrolyte balance and Wnt signaling during stem cell and embryo development [64, 65]. The fusion of synaptic vesicles with the presynaptic membrane in neurons necessitates the isolation of the V1 and V0 subunits; meanwhile, the mechanisms coordinating these events remain elusive [66]. Notably, this phenomenon is not exclusive to the previously mentioned subunits but also manifests in the context of ATP6AP1. The auxiliary subunit ATP6AP1 acts as a key regulator of V-ATPase that regulates and activates V-ATPase, consequently adjusting the intracellular pH [21]. Moreover, ATP6AP1 affects lysosomal trafficking and exocytosis [23, 67]. ATP6AP1 may be responsible for intragranular acidification in the endocrine pancreas. Our study demonstrated that ATP6AP1 promotes the fusion of autolysosomes by facilitating HOPS tethering to Rab7 (Fig. 5).

In nutrient signaling, AMP-activated protein kinase (AMPK) and the mechanistic target of rapamycin complex 1 (mTORC1) play crucial roles in maintaining cellular energy homeostasis by monitoring cellular levels of ATP and nutrients, such as glucose and amino acids [68]. Recent studies have revealed that V-ATPases regulate the activities of mTOR and AMPK pathways. Efeyan et al. found that V-ATPase senses high nutrient and energy levels and stimulates the GEF activity of Ragulator, thereby increasing the affinity of RAGs for mTORC1 [69]. Zhang et al. reported that the late endosomal/lysosomal protein complex V-ATPase-Ragulator, essential for activation of mTORC1, is also required for AMPK activation [70]. Therefore, we hypothesize that ATP6AP1 might also serve as a key regulator of AMPK and mTORC1 by regulating V-ATPase activity, which requires more in-depth studies in the future.

**Fig. 7:**
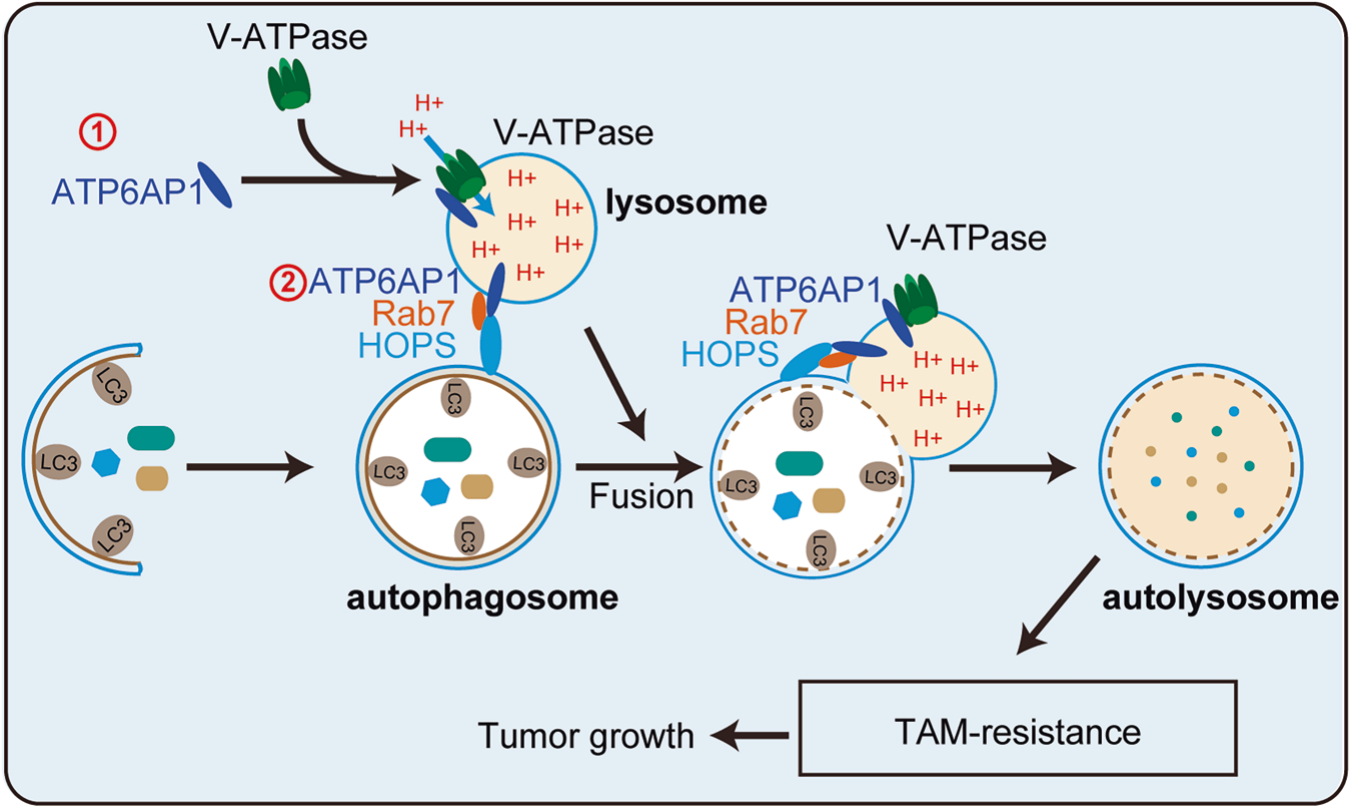
Working model. The Schematic shows how ATP6AP1 promotes cell growth and TAM resistance in luminal breast cancer cells by inducing autophagy.

In summary, our study demonstrated that ATP6AP1 accelerates the process of autophagy by facilitating V-ATPase-mediated lysosomal acidification and Rab7-dependent lysosome fusion, therefore promoting cell proliferation and drug resistance in luminal breast cancer cells (Fig.7). ATP6AP1 could serve as both a specific biomarker and a potential therapeutic target for luminal breast cancer.

## Materials and methods

### Antibodies, reagents, and plasmids

The antibodies used in this study include anti-p62 (Cell Signaling Technology, 5114S), anti-LC3B (Cell Signaling Technology, 3868S), anti-HA (Cell Signaling Technology, 3724S), anti-ATP6V0D1 (Proteintech, 18274-1-AP), anti-ATP6V1B2 (Proteintech, 15097-1-AP), anti-LAMP2 (Proteintech, 66301-1-Ig), anti-ATG5 (Proteintech, 10181-2-AP), anti-LC3B (Abcam, ab192800), anti-Rab7 (Abcam, ab137029), anti-GAPDH (Millipore, MAB374), anti-Flag (Sigma‒Aldrich, F3165), anti-GFP (Sigma‒Aldrich, G1544), anti-ATP6AP1 (Santa Cruz, sc-81886), normal rabbit IgG (Santa Cruz, sc-2027), goat anti-rabbit IgG (Thermo Fisher Scientific, 31460), goat anti-mouse IgG (Thermo Fisher Scientific, 31430).

Chemical reagents and assay kits include EBSS (Invitrogen, 14155063), Chloroquine (Sigma‒Aldrich, C6628), dimethyl sulfoxide (DMSO; Sigma‒Aldrich,D2650), protease inhibitor cocktail (Sigma‒Aldrich, P8340), and polyethyleneimine (PEI) transfection reagent (Sigma‒Aldrich, 408727), Puromycin (Solarbio, P8230), Lipofectamine 2000 transfection reagent (Thermo Fisher Scientific, 11668019), TRIzol reagent (Thermo Fisher Scientific, 15596018), PrimeScript RT Reagent Kit with gDNA Eraser (Takara Bio, RR047A), SYBR Premix Ex Taq Tli RNaseH Plus (Takara Bio, RR820A), Tamoxifen (MedChemExpress, HY-13757A), Bafilomycin A1 (MedChemExpress, HY-100558), LysoSensorTM Green DND-189 (YEASEN, 40767ES50), Lysosome Isolation Kit (Abcam, ab234047), Protein A-agarose beads (Roche, 11134515001) and Protein G-agarose beads (Roche, 11243233001), The Cell-light EdU (5-ethynyl-2′-deoxyuridine) Apollo 567 In Vitro Kit (RiboBio Biology, C10310–1), Cell-light EdU Apollo 488 In Vitro Kit (RiboBio Biology, C10310–3), A Cell Counting Kit 8 (CCK-8; Beyotime, C0038), Annexin V-allophycocyanin (APC)/7-AAD Apoptosis Kit (Keygen, KGA1024). The other analytical-grade chemicals were purchased from Sigma‒Aldrich.

The human full-length ATP6AP1 (NM_001183) construct was cloned into pEZ-Lv242 (GeneCopoeia, Rockville, MD). The shRNA for ATP6AP1 was cloned into the shRNA expression vector psi-LVRU6MP (GeneCopoeia, Rockville, MD). The human full-length VPS39 (NM_001301138) and Rab7 (NM_004637) constructs were cloned into pEGFP-C3 (Clontech Laboratories, Mountain View, CA) and pKH3, respectively. The plasmids for pLVX-mCherry-EGFP-LC3B were purchased from Miaolingbio (Wuhan, China). The target sequences of the shRNA mentioned above expression constructs are listed in Table S1.

### Cell culture and transfection

The transformed human embryonic kidney cell line HEK-293T and human luminal A breast cancer cell lines (MCF-7, ZR-75-1 and T47D), human luminal B breast cancer cell lines (BT-474) were purchased from the American Type Culture Collection (ATCC, Manassas, VA, USA). ZR-75-1 and T47D cells were cultured in RPMI 1640 medium (Gibco, C11875500BT) containing 10% fetal bovine serum (Gibco, 10099-141) and antibiotics (100 U/ml streptomycin and 100 μg/ml penicillin; Invitrogen, 15140-122), BT-474 cells were cultured in RPMI 1640 medium containing 15% fetal bovine serum and antibiotics (100 U/ml streptomycin and 100 μg/ml penicillin). HEK-293T and MCF-7 cells were cultured in Dulbecco’s modified Eagle’s medium (DMEM; Gibco, C11995500BT) containing 10% fetal bovine serum and antibiotics (100 U/ml streptomycin and 100 μg/ml penicillin). Cell culture was performed in a humidified incubator with 5% CO_2_ at 37°C. These cells were authenticated using short tandem repeat (STR) profiling, and no mycoplasma contamination was found via PCR-based assay (performed in August 2019). The used cells were immediately expanded and frozen to be resuscitated every 3 to 4 months from a frozen vial of the same batch of cells.

Cells were transiently transfected with Lipofectamine 2000 for luminal breast cancer cell lines or with polyethyleneimine (PEI) for HEK-293T cells according to the manufacturer’s instructions. In all experiments, the medium was replaced daily. All stable cell lines infected with lentivirus harboring ATP6AP1 overexpression or knockdown constructs were treated with 0.5-1 μg/ml puromycin for 3 days, and selected clones were confirmed as positive by both PCR and Western blotting.

### Western blotting and quantitative real-time PCR

Cells were harvested and lysed in radioimmunoprecipitation (RIPA) lysis buffer (50 mM Tris-HCl, pH 7.4, 150 mM NaCl, 1% Nonidet P-40, 0.1% sodium dodecyl sulfate, and protease inhibitor). Cell lysates were cleared by centrifugation at 10,000 × g for 10 min. The protein concentrations were measured using the bicinchoninic acid (BCA) method. Cell lysates were separated by 12% sodium dodecyl sulfate-polyacrylamide gel electrophoresis (SDS-PAGE) and transferred onto nitrocellulose (NC) membranes (Millipore, HATF00010). After blocked with 5% skim milk and washed in Tris Buffered Saline with Tween pH 7.6 (TBST), the blots were incubated with antibodies at 4°C overnight and then incubated with anti-rabbit/mouse IgG. Bands were exposed by chemiluminescence and quantified using ImageJ software (National Institutes of Health, Bethesda, MD, USA).

Total RNA was extracted using TRIzol reagent, and 1 μg was used to prepare cDNA by reverse transcription using the PrimeScript RT Reagent Kit with gDNA Eraser. Quantitative real-time PCR (RT‒qPCR) was carried out on an ABI StepOnePlus Real-Time PCR System (Applied Biosystems, Foster City, CA, USA) using SYBR Premix Ex Taq Tli RNaseH Plus, and the primers are listed in Table S2. The variation trend in expression levels of target genes was normalized by GAPDH RNA levels as an internal control. Data are presented as the mean ± SD of at least three independent experiments.

### Cell proliferation assay

The cell proliferation rate was determined by a cell growth curve after counting cell numbers. The lentivirus-infected breast cancer stable cells were seeded in 24-well plates at 2000 cells/well density and incubated in a cell incubator with 5% CO_2_ at 37°C. Cell numbers were counted by flow cytometry daily for 7 days and the cell culture medium was refreshed every 2-3 days depending on the consumption.

### CCK-8 assay

A Cell Counting Kit 8 (CCK-8) assay was employed to determine the viability of luminal breast cancer cells. In general, a density of 5 × 10^3^ cells were seeded into 96-well plates, followed by treatment with different concentrations of tamoxifen for 72 h. Then, CCK-8 solution was added to each well according to the manufacturer’s instruction and incubated for the indicated time to determine the absorbance values of each well. The OD values between the control and treated groups examined at 450 nm with a microplate reader.

### Apoptosis assay

Cell apoptosis was measured using an annexin V-allophycocyanin (APC)/7-AAD Apoptosis Kit. T47D cells stably infected with LV-ATP6AP1 were seeded into 6-well plates and treated with DMSO, 5 μM TAM, or 5 μM TAM + 10 μM CQ for 48 h. Cells were collected and washed with PBS. After adding 500 μl 1x Annexin V Binding Buffer, the pellets were incubated in, 5 μl 7-AAD, 5 μl annexin V-APC for 20 min at room temperature in the dark. Samples were analyzed with an Accuri C6 Plus flow cytometer (BD, NJ, USA).

### EdU-DNA synthesis assays

Lentivirus-infected BRCA cells were seeded in 96-well plates at a density of 8 × 10^3^/well. The cell culture medium was replaced with 50 μM EdU solution diluted in the growth culture medium after 24 h, followed by incubation for 2 h. The cells were then processed with the Cell-light EdU Apollo 567/488 In Vitro Kit according to the manufacturer’s instructions. In short, cells were fixed with 4% paraformaldehyde for 30 min and then washed with PBS. After that, cells were incubated with 1X Apollo 488/567 Dye reaction solution and 1X Hoechst 33342 Dye reaction solution respectively for 30 min at room temperature in the dark. Images were acquired on an inverted fluorescence microscope (IX71; Olympus, Tokyo, Japan) and analyzed with ImageJ software (National Institutes of Health, Bethesda, MD, USA).

### Xenograft mouse model

For in vivo experiments, 5 × 10^6^ ZR-75-1 cells stably infected with LV-control and LV-ATP6AP1 were suspended in PBS with 10% Matrigel and then injected into the flanks of 4-week-old female BALB/c-nu athymic nude mice (SLAC Laboratory Animal Co., Hunan, China; *n* = 6 mice per group). When tumors reached a volume of ~100 mm^3^, we randomly allocated the mice to groups receiving a placebo or the indicated drugs. Tamoxifen (TAM) was administered at a dose of 30 μg per mouse every 2 days. At 24 days after injection, tumors were harvested and weighted. Protocols for animal experiments were approved by the Ethical Committee of the First Affiliated Hospital of Nanchang University and conformed to the guidelines of the National Institutes of Health on the ethical use of animals.

### Lysosomal pH assay

Lysosomal pH was measured using the fluorescent acidotropic probe LysoSensor Green DND-189 (Invitrogen, USA), which would show the fluorescence intensity under acidification. The lentivirus-infected breast cancer stable cells were seeded in 6-well plates. After 24 h until the cells raised at proper density, LysoSensor Green was added directly to the medium at a final concentration of 5 mM and incubated for 2 h at 37 °C with 5% CO_2_. The control group was added the medium without probe instead in the same situation. Cells were collected and washed with PBS, and then the pellets were dissolved in PBS and analyzed with an Accuri C6 Plus flow cytometer (BD, NJ, USA).

### Lysosome Isolation

To examine the expression of V-ATPase subunits and Rab7 in lysosomes, we purified lysosomes using a Lysosomal Isolation kit. We isolated 2 × 10^7^ T47D cells with 500 μl Lysosome Isolation Buffer. Homogenize the cells dozens of times with a glass Dounce homogenizer on ice, and add 500 μl lysosome enrichment buffer. Centrifuge at 500 x g for 10 min at 4°C. Collected the supernatant and mixed with 200 μl Density Gradient Media II/Lysosome Gradient. Discontinuous Density Gradient Solutions were prepared using Density Gradient Media II/Lysosome Gradient & Lysosome Enrichment Gradient Solutions. Slowly tiling was started from the high-density gradient solution and the gradient solution was successively tiled inside the centrifuge tube. Finally, the diluted cell lysate was slowly spread on a discontinuous density gradient. Ultracentrifuging was performed for 2 h at 145,000 x g at 4°C using an ultracentrifuging tube (326819, Beckman) and a matching rotor (MLS-50, Beckman). The lysosome band is visible in the top 1/10th of the gradient volume and about 0.5 ml of the lysosome fraction band was extracted from the top of the gradient. Mix this fraction with 1 ml PBS and centrifuge for 30 min at 16,900 x g at 4°C. The supernatant was discarded to obtain the purified lysosomal pellet. The Bradford method was used to determine the concentration of lysosomal proteins, and western blot was used to detect the expression of corresponding proteins.

### Immunofluorescence

To detect endogenous LC3B and LAMP2, about 5×10^3^-2×10^4^ lentivirus-infected breast cancer stable cells were seeded on coverslips in 24-well plates. Cells were incubated with EBSS for 4 h. Then, the cells were washed with PBS 3 times, fixed with 4% polyformaldehyde for 30 min, incubated with 0.3% Triton X-100 for 10 min, blocked in PBS containing 5% BSA for 30 min, and labeled with the indicated primary antibodies (LC3B, 1:200; LAMP2, 1:200) at 4°C overnight. After washed 3 times with PBS, cells were incubated with the indicated secondary antibodies at room temperature for 1 h. Nuclei were stained with DAPI for 10 min. Images were acquired as a confocal Z-stack with 8 μm spacing using a confocal microscope (LSM 700, Zeiss).

### Patients and clinical samples

Human primary breast specimens of luminal breast cancer tissues and the corresponding adjacent tissues, as well as paraffin-embedded tissue blocks, were obtained from the Department of Pathology and a tissue bank at The First Affiliated Hospital of Nanchang University, China. Samples were reviewed by a pathologist to ensure that they included tumors and adjacent tissue. The histologic type was defined according to the World Health Organization classification criteria. Specimens were collected and processed in compliance with protocols approved by the Institutional Review Board of Nanchang University. All patients signed an informed consent form.

### Immunohistochemistry

Immunohistochemical staining was performed as described previously [71]. Immunohistochemistry staining for ATP6AP1 was performed on paraffin-embedded luminal breast cancer tissue and adjacent tissue microarrays. Briefly, 3 μm thick paraffin sections were dewaxed, rehydrated, and repaired in 10 mM sodium citrate buffer (pH 6.0) under high pressure for 2.5 min and incubated in 3% hydrogen peroxide solution for 10 min after being washed with PBS 3 times. Normal goat serum (10%) was used to block nonspecific staining, and then the tissue sections were exposed to the indicated antibodies. The immunoreactivity of the tissues was captured under the FSX100 microscope equipped with a digital camera system (Olympus) after incubation with Polink-2 HRP DAB Detection Kit under specification. The stained sections were observed by at least two independent investigators blinded to the histopathological features of the samples. The German semiquantitative scoring system was employed to assess the staining intensity and stained area.

### Statistical analysis

The data represent the mean ± SD values of samples obtained from three independent experiments. We performed a two-tailed Student’s *t*-test to determine statistically significant differences between the two groups. Differences between multiple groups were determined by Dunnett’s multiple comparison test. The Kaplan‒Meier method was employed to plot survival curves, and differences were calculated with the log-rank test. In the statistical analysis, *p* < 0.05 was deemed statistically significant.

## Supplementary information

Supplementary Information

Original western blots

## Data Availability

All data generated or analyzed are included in the article and its supplementary files, and available from the corresponding author upon request.
