# Supplementary material for: ATP6AP1 promotes cell proliferation and tamoxifen resistance in luminal breast cancer by inducing autophagy
Source: Cell Death Dis. 2025 Mar 25;16(1):201. doi: 10.1038/s41419-025-07534-y (PMC11937278; doi:10.1038/s41419-025-07534-y)
Supplement: Supplementary file 1 — Supplementary Information [file 41419_2025_7534_MOESM1_ESM.pdf]

Figure S1

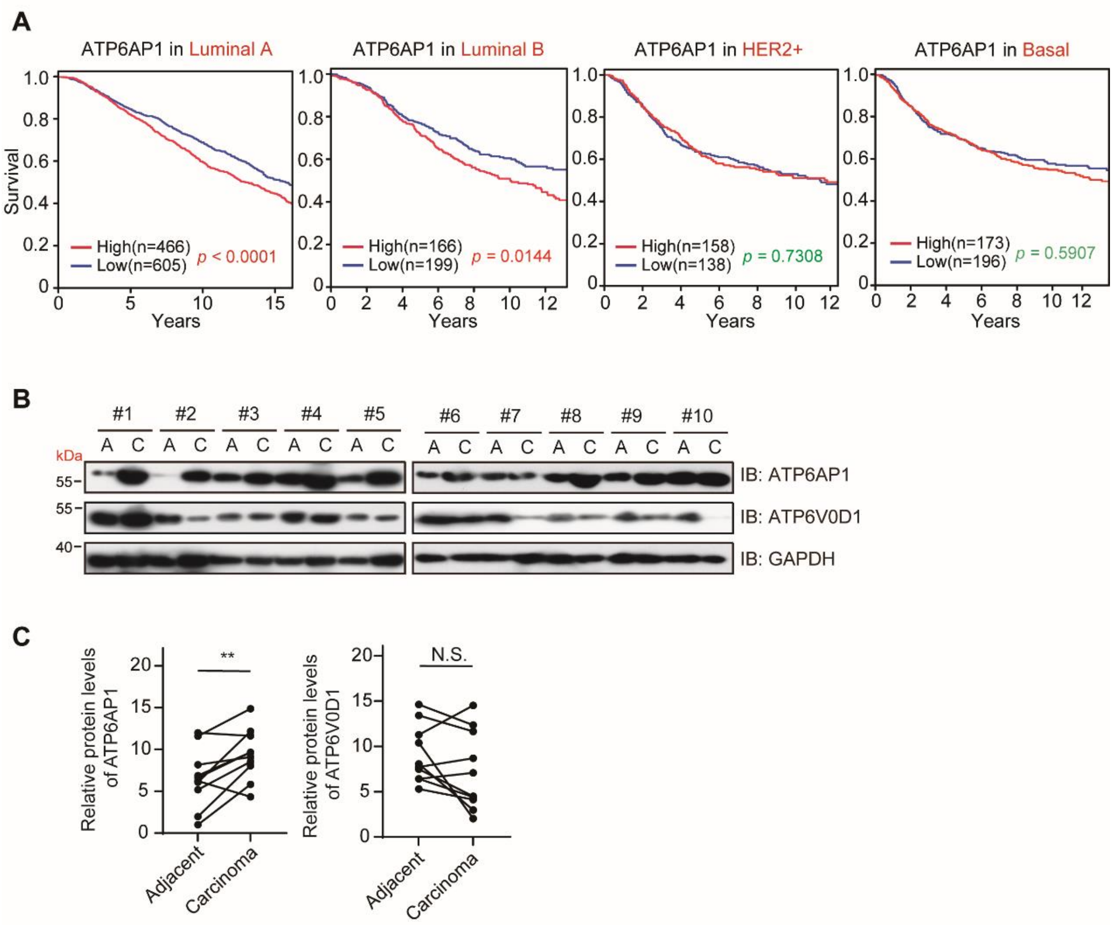

Figure S1. Supplementary figure to Figure 1

(**A**) Kaplan–Meier survival curve analysis of the prognostic significance of high and low expression of ATP6AP1 in different subclasses of breast cancer using the bc-GenExMiner tool.

(**B and C**) Western blotting analysis of ATP6AP1 and ATP6V0D1 expression in luminal breast cancer samples and matched adjacent breast tissue samples. A, adjacent tissue; C, carcinoma tissue. n = 10, ***p* <0.01, N.S., no significance.

Figure S2

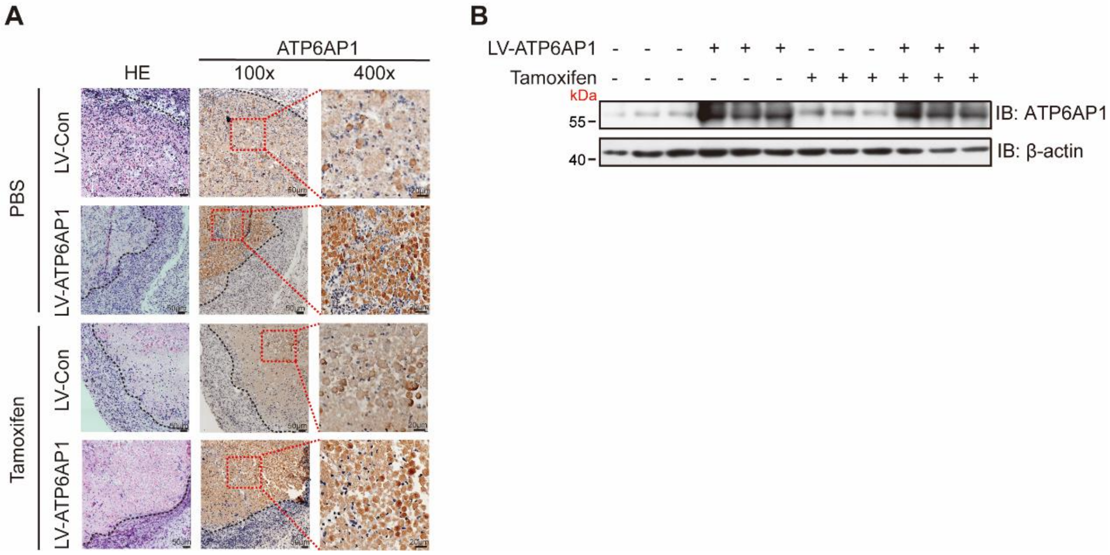

Figure S2. Supplementary figure to Figure 2

(**A and B**) Xenograft mouse models were developed using ZR-75-1 cells that stably overexpressd ATP6AP1 or contain an empty vector. The overexpression of ATP6AP1 was verified by IHC **(A)** and western blotting **(B)**. Scale bars, 50 μm (left), 20 μm (right).

Figure S3

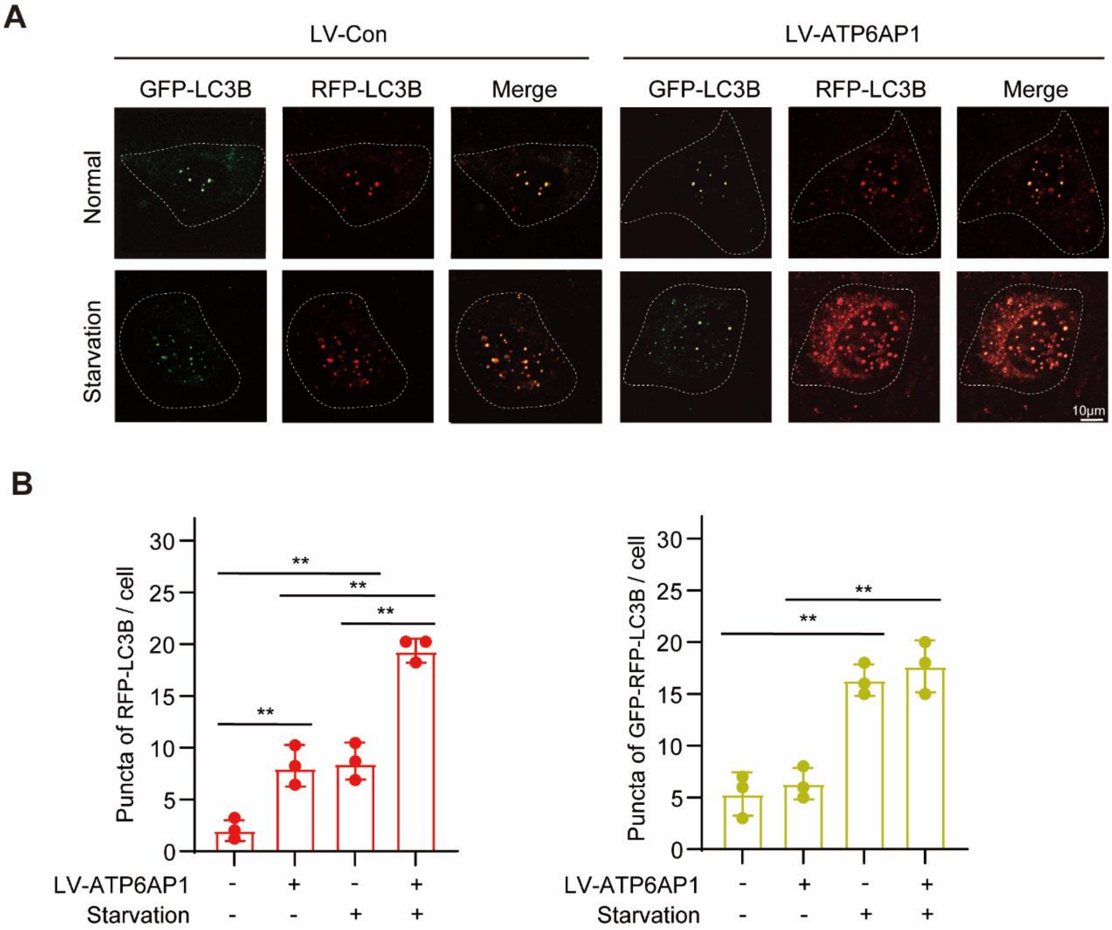

Figure S3. Supplementary figure to Figure 4

(**A**) T47D cells were transfected with LV-Con or LV-ATP6AP1 for 48 h and then treated with HBSS for 6 h. Green and red fluorescence was detected by confocal microscopy. Scale bars, 10 μm.

(**B**) Quantification of autophagic flux as analyzed in (**A**). The average percentage of yellow puncta and red puncta per cell was calculated. The bar graph displays the mean±SD, n=3, ***p*<0.01.

Figure S4

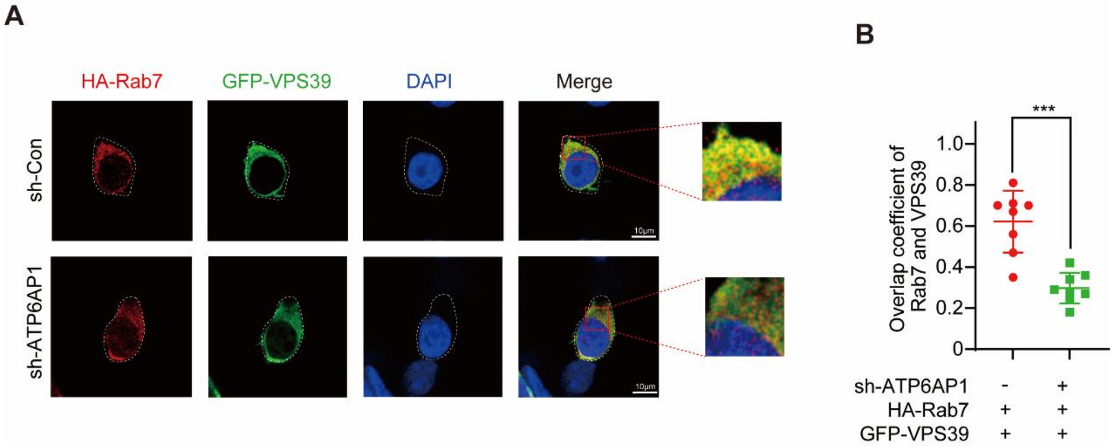

Figure S4. Supplementary figure to Figure 5

(**A**) The knockdown of ATP6AP1 reduces the co-localization of Rab7 with VPS39. Representative immunofluorescence images showing the co-localization of Rab7 and VPS39 in MCF-7 cells that were infected with Lv-sh-ATP6AP1 and transfected with HA-Rab7 and GFP-VPS39. Scale bars, 10 μm.

(**B**) Quantification of Rab7-VPS39 colocalized voxels (volumetric pixels). Mean±SD, ****p*< 0.001 by two-way ANOVA (n =8).

Figure S5

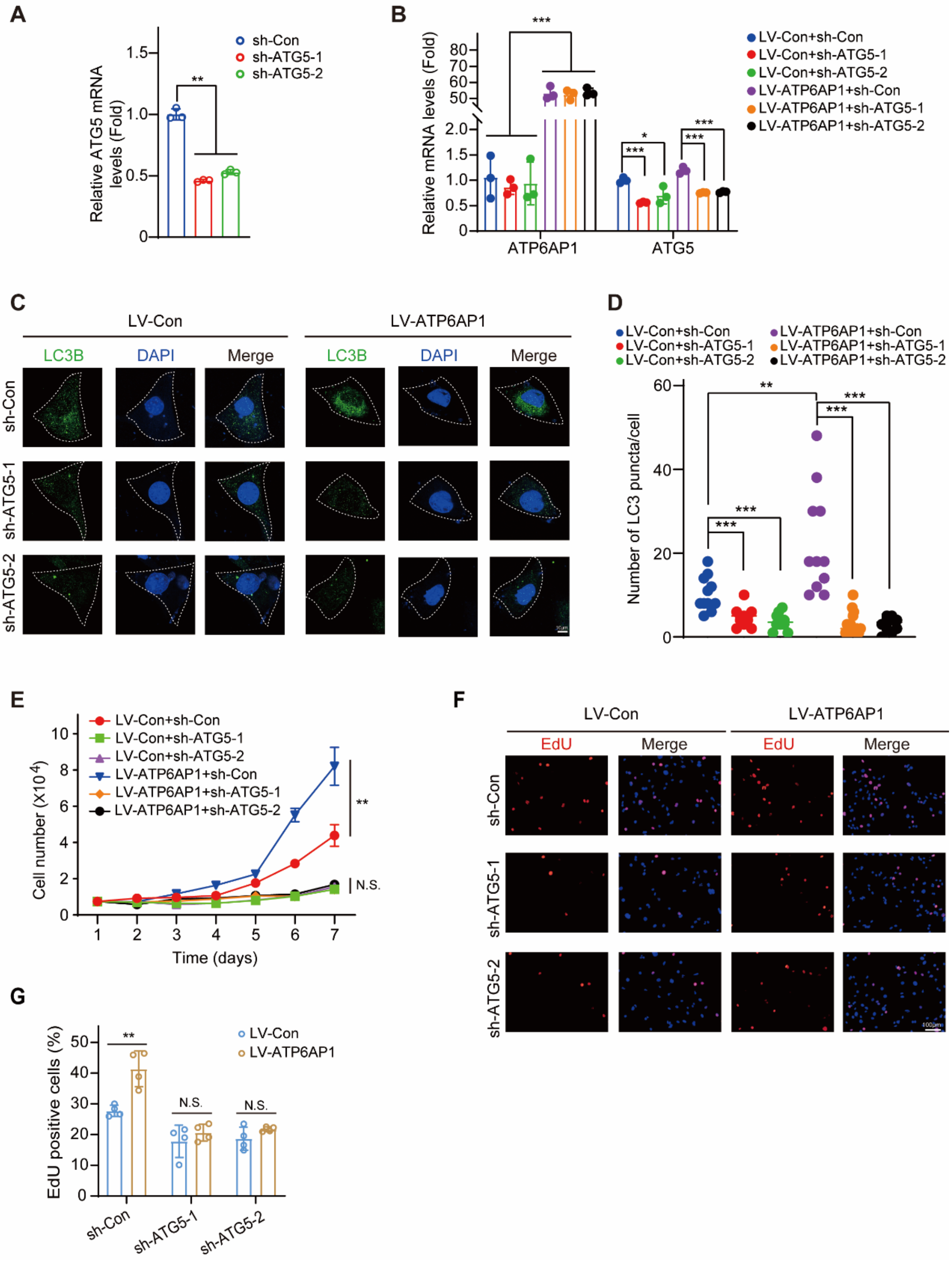

Figure S5. Supplementary figure to Figure 6

(**A**) RT–qPCR was used to verify the interference efficiency of ATG5 shRNA in ZR-75-1 cells. The bar graph displays the mean±SD, n=3, **p<0.01.

(**B**) T47D cells were infected with LV-ATP6AP1 and LV-sh-ATG5 lentiviruses

and harvested for RT–qPCR. The bar graph displays the mean±SD, n=3, *p<0.05, ***p<0.001.

(**C**) Knocking down ATG5 attenuated ATP6AP1- increased LC3 puncta. T47D cells infected with LV-ATP6AP1 or LV-sh-ATG5 were treated with EBSS for 4 hours. Representative immunofluorescence images of LC3 puncta were shown. Scale bars, 10μm.

(**D**) Quantification of LC3 puncta (volumetric pixels). Mean±SD, *** $p < 0.001$, ** $p < 0.01$ by two-way ANOVA (n=10).

(**E**) ATP6AP1 facilitates luminal breast cancer cell proliferation by activating autophagy. T47D cells were infected with ATP6AP1 overexpression (LV-ATP6AP1) and ATG5 knockdown (LV-sh-ATG5) lentiviruses. Cell proliferation was determined by flow cytometry. The graph displays the mean±SD, n=3, **p<0.01, N.S., no significance.

(**F**) Cell proliferation of T47D cells was determined by EdU assay. Scale bars, 100 μm.

(**G**) Quantification of the data in (**F**). The bar graph displays the mean±SD, n=3, **p<0.01, N.S., no significance.

Supplementary Table 1: shRNAs used for knockdown of specific genes

	Target sequences (5’-3’)	Start
shRNA-ATP6AP1(#1)	5’-GCAGCTCTCTACCTACTTAGA-3’	232
shRNA-ATP6AP1(#2)	5’-ACAGTGACATTCAAGTTCATT-3’	974
shRNA-ATP6AP1(#3)	5’-GTCGCCTACTTCAATGCTTCC-3’	1073
shRNA-ATG5(#1)	5’-GATTCATGGAATTGAGCCAAT-3’	1010
shRNA-ATG5(#2)	5’-CAGGATGAGATAACTGAAAGG-3’	363

Supplementary Table 2: Primers used for real-time PCR analyses

ATP6AP1	Forward primer (5’ to 3’)	5’-CTTCTGGAATGACTCCTTTGCC-3’
	Reverse primer (5’ to 3’)	5’-ATTGCTGTGGACTTCGAGG-3’
ATG5	Forward primer (5’ to 3’)	5’-AAAGATGTGCTTCGAGATGTGT-3’
	Reverse primer (5’ to 3’)	5’-CACTTTGTCAGTTACCAACGTCA-3’
GAPDH	Forward primer (5’ to 3’)	5’-CACCAGGGCTGCTTTTAACTCTG-3’
	Reverse primer (5’ to 3’)	5’-GATTTTGGAGGGATCTCGCTCCTG-3’
