# Supplementary material for: ATP6AP1 promotes cell proliferation and tamoxifen resistance in luminal breast cancer by inducing autophagy
Source: Cell Death Dis. 2025 Mar 25;16(1):201. doi: 10.1038/s41419-025-07534-y (PMC11937278; doi:10.1038/s41419-025-07534-y)

Fig. 2B ZR-75-1

IB:Flag

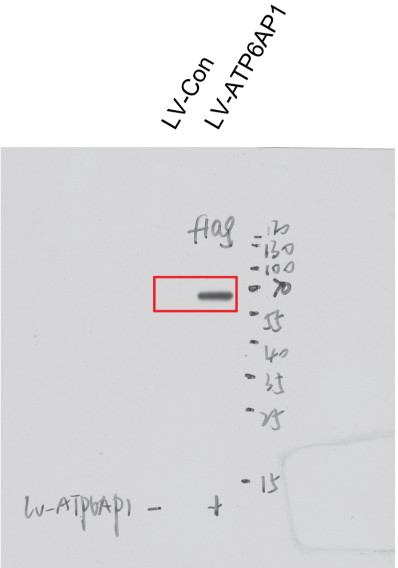

IB:GAPDH

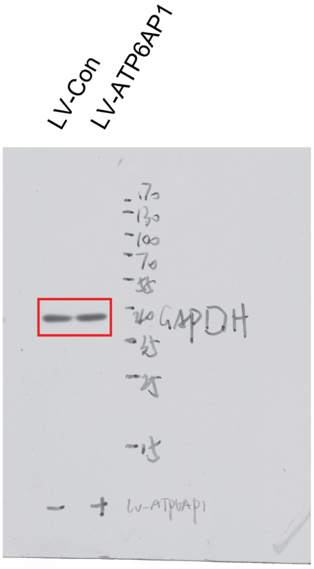

Fig. 2B T47D

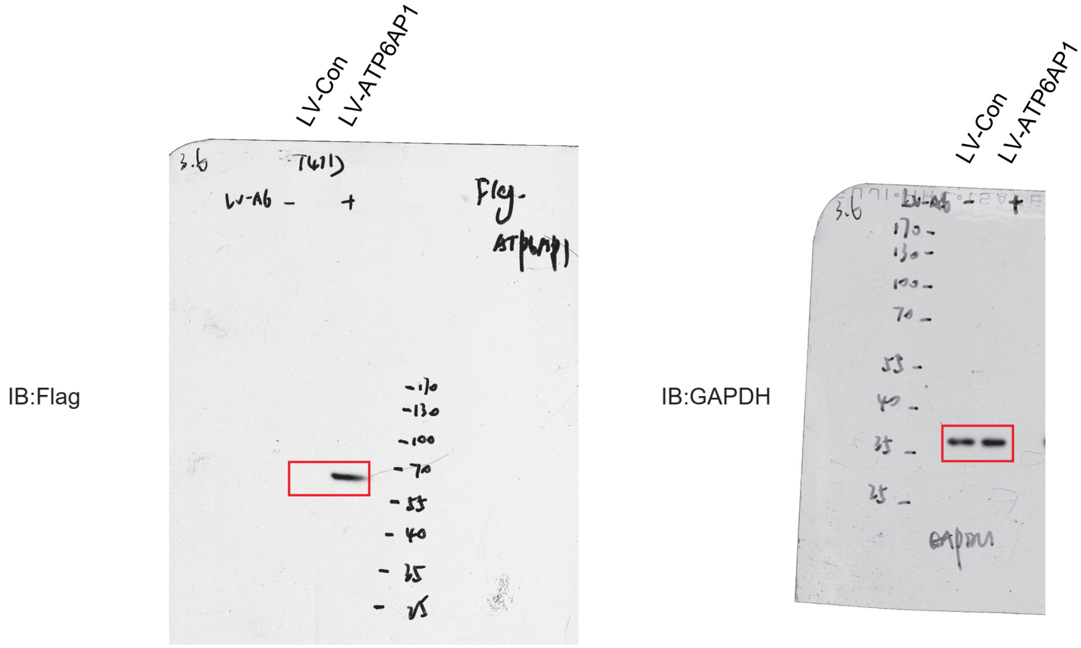

Fig. 2B MCF-7

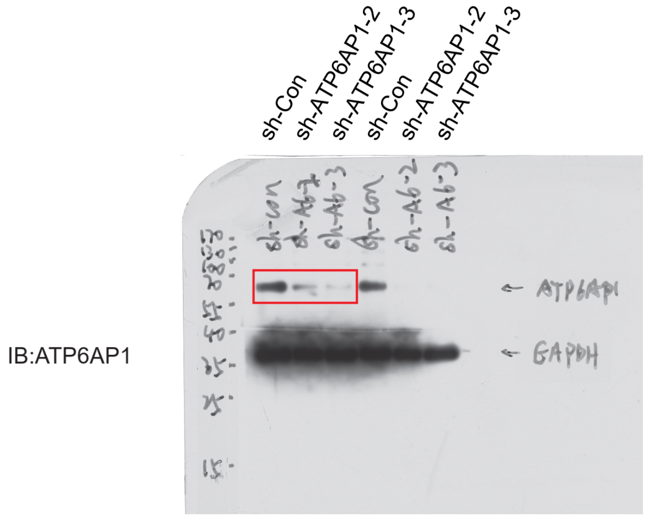
sh-Con
sh-ATP6AP1-2
sh-ATP6AP1-3
sh-Con
sh-ATP6AP1-2
sh-ATP6AP1-3
IB:ATP6AP1
ATP6AP1
GAPDH

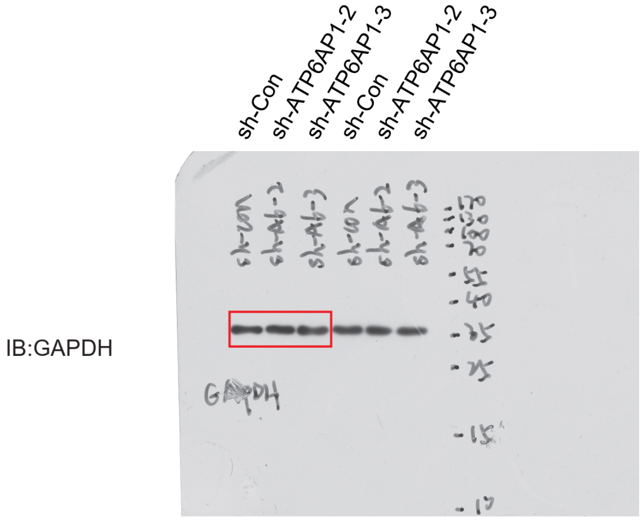
sh-Con
sh-ATP6AP1-2
sh-ATP6AP1-3
sh-Con
sh-ATP6AP1-2
sh-ATP6AP1-3
IB:GAPDH
GAPDH

Fig. 2B BT-474

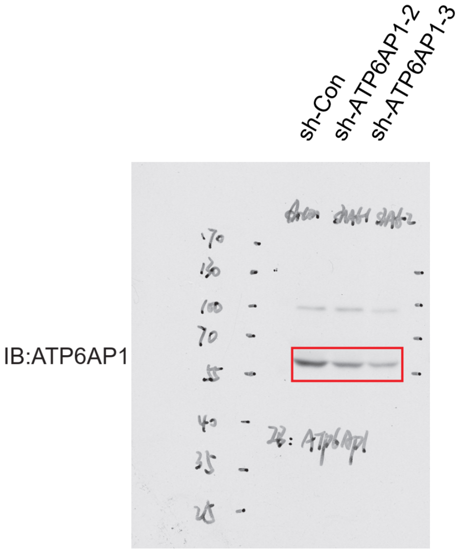
sh-Con
sh-ATP6AP1-2
sh-ATP6AP1-3
IB:ATP6AP1

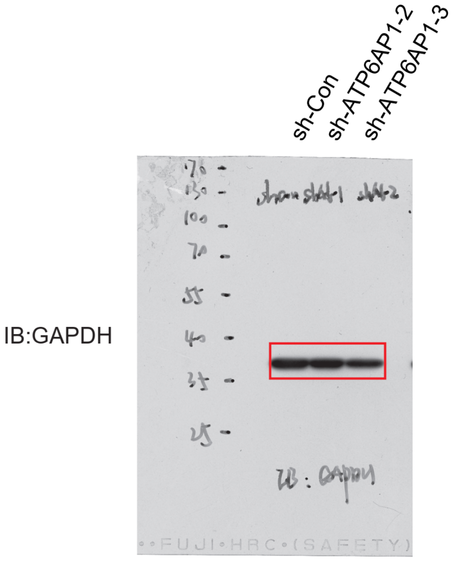
sh-Con
sh-ATP6AP1-2
sh-ATP6AP1-3
IB:GAPDH

Fig. 3A

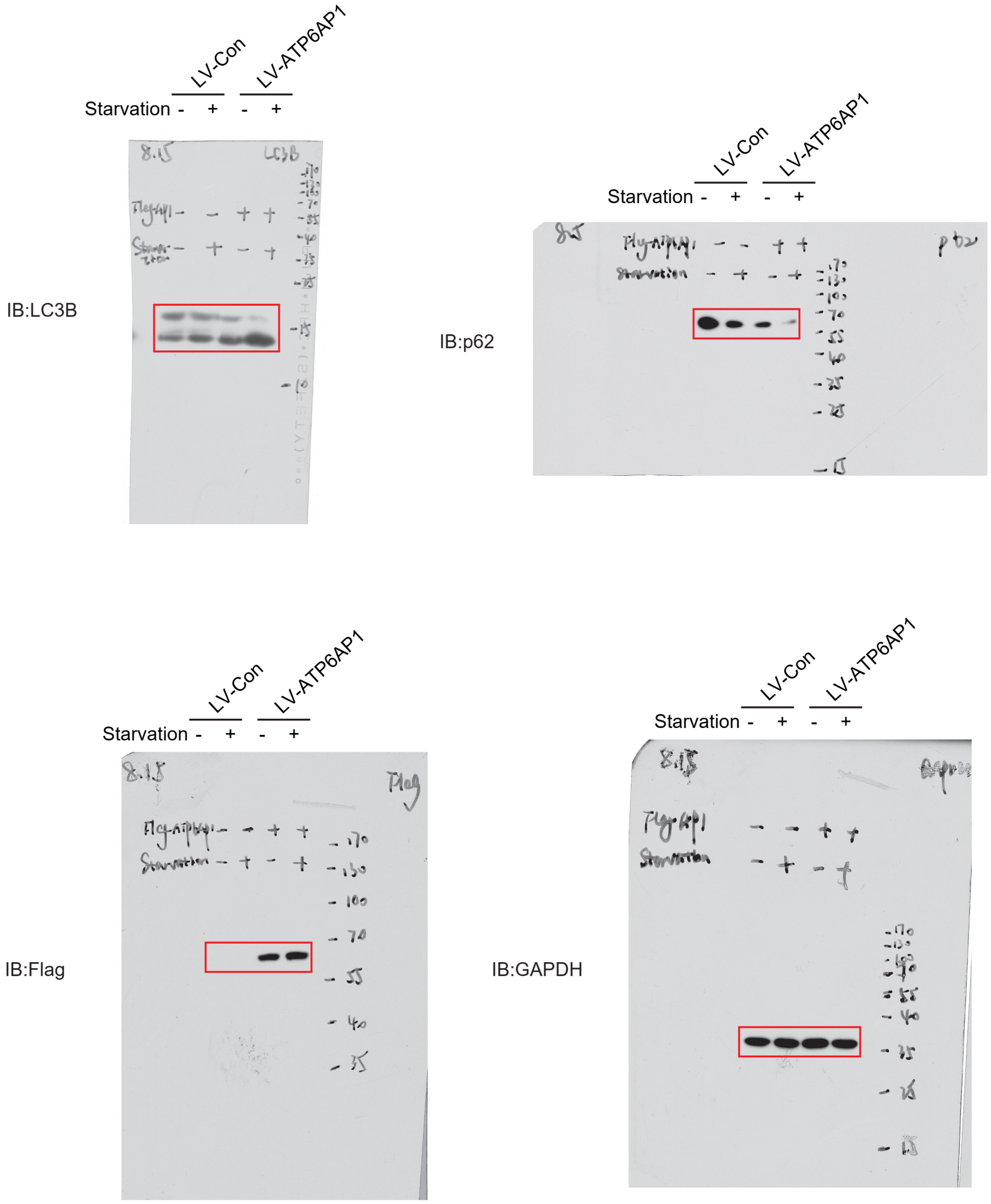
LV-Con
LV-ATP6AP1
Starvation - + - +
IB:LC3B
LV-Con
LV-ATP6AP1
Starvation - + - +
IB:p62
LV-Con
LV-ATP6AP1
Starvation - + - +
IB:Flag
LV-Con
LV-ATP6AP1
Starvation - + - +
IB:GAPDH

Fig. 3D

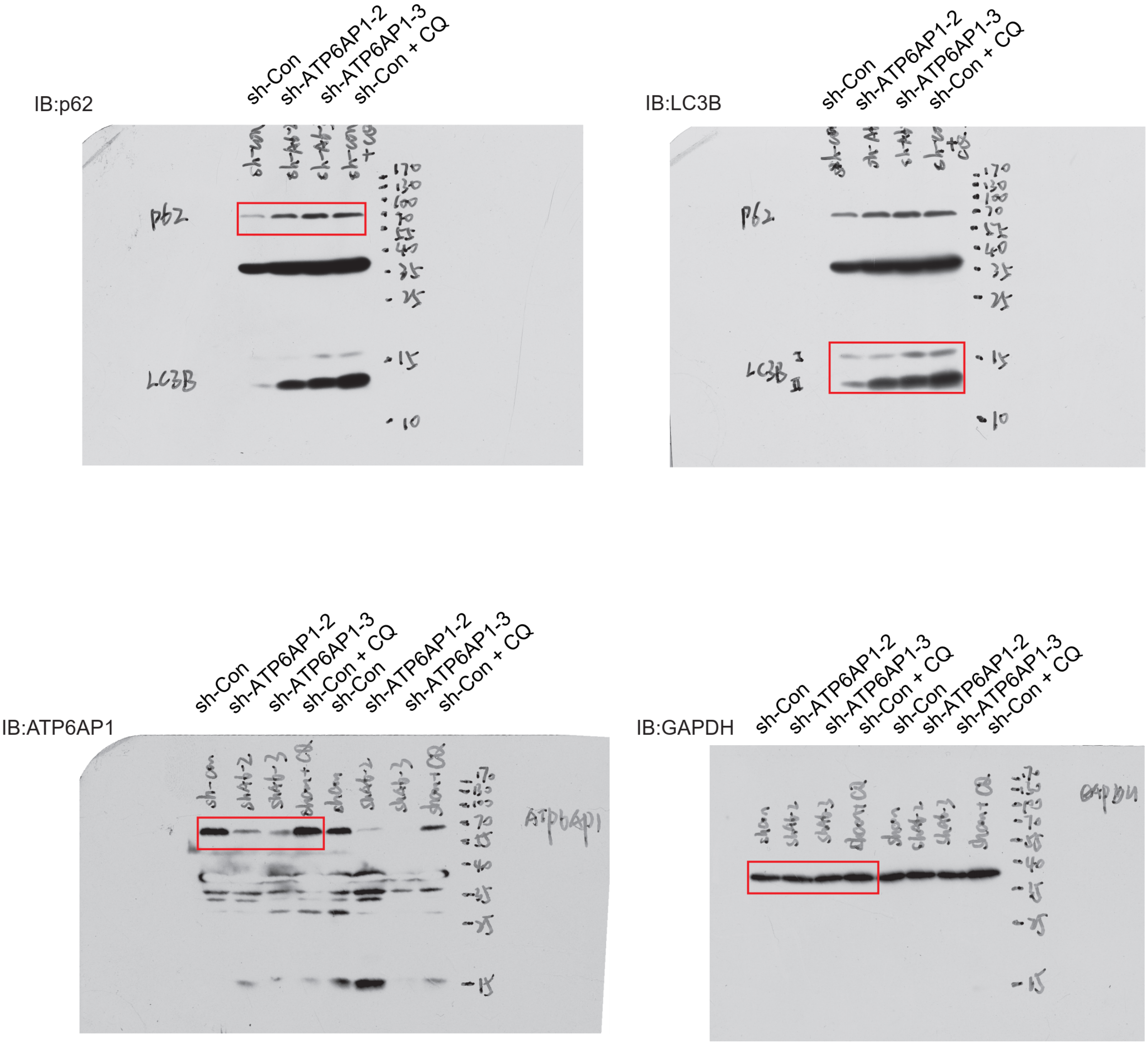
IB:p62
sh-Con
sh-ATP6AP1-2
sh-ATP6AP1-3
sh-Con + CQ
IB:LC3B
sh-Con
sh-ATP6AP1-2
sh-ATP6AP1-3
sh-Con + CQ
IB:ATP6AP1
sh-Con
sh-ATP6AP1-2
sh-ATP6AP1-3
sh-Con + CQ
sh-Con
sh-ATP6AP1-2
sh-ATP6AP1-3
sh-Con + CQ
IB:GAPDH
sh-Con
sh-ATP6AP1-2
sh-ATP6AP1-3
sh-Con + CQ
sh-Con
sh-ATP6AP1-2
sh-ATP6AP1-3
sh-Con + CQ

Fig. 4F

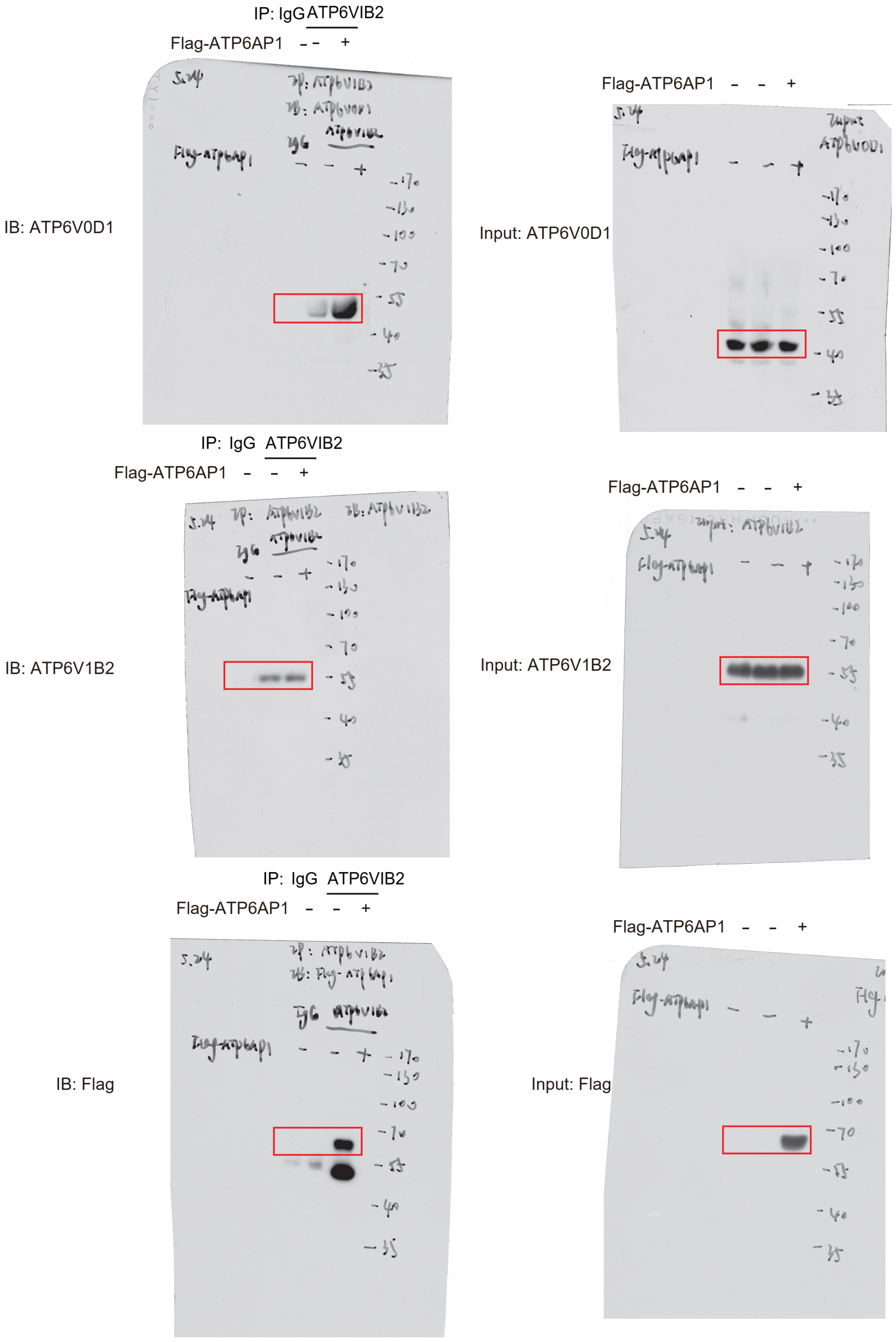
IP: IgG ATP6VIB2
Flag-ATP6AP1 - - +
IB: ATP6V0D1
Flag-ATP6AP1 - - +
Input: ATP6V0D1
IP: IgG ATP6VIB2
Flag-ATP6AP1 - - +
IB: ATP6V1B2
Flag-ATP6AP1 - - +
Input: ATP6V1B2
IP: IgG ATP6VIB2
Flag-ATP6AP1 - - +
IB: Flag
Flag-ATP6AP1 - - +
Input: Flag

Fig. 4G

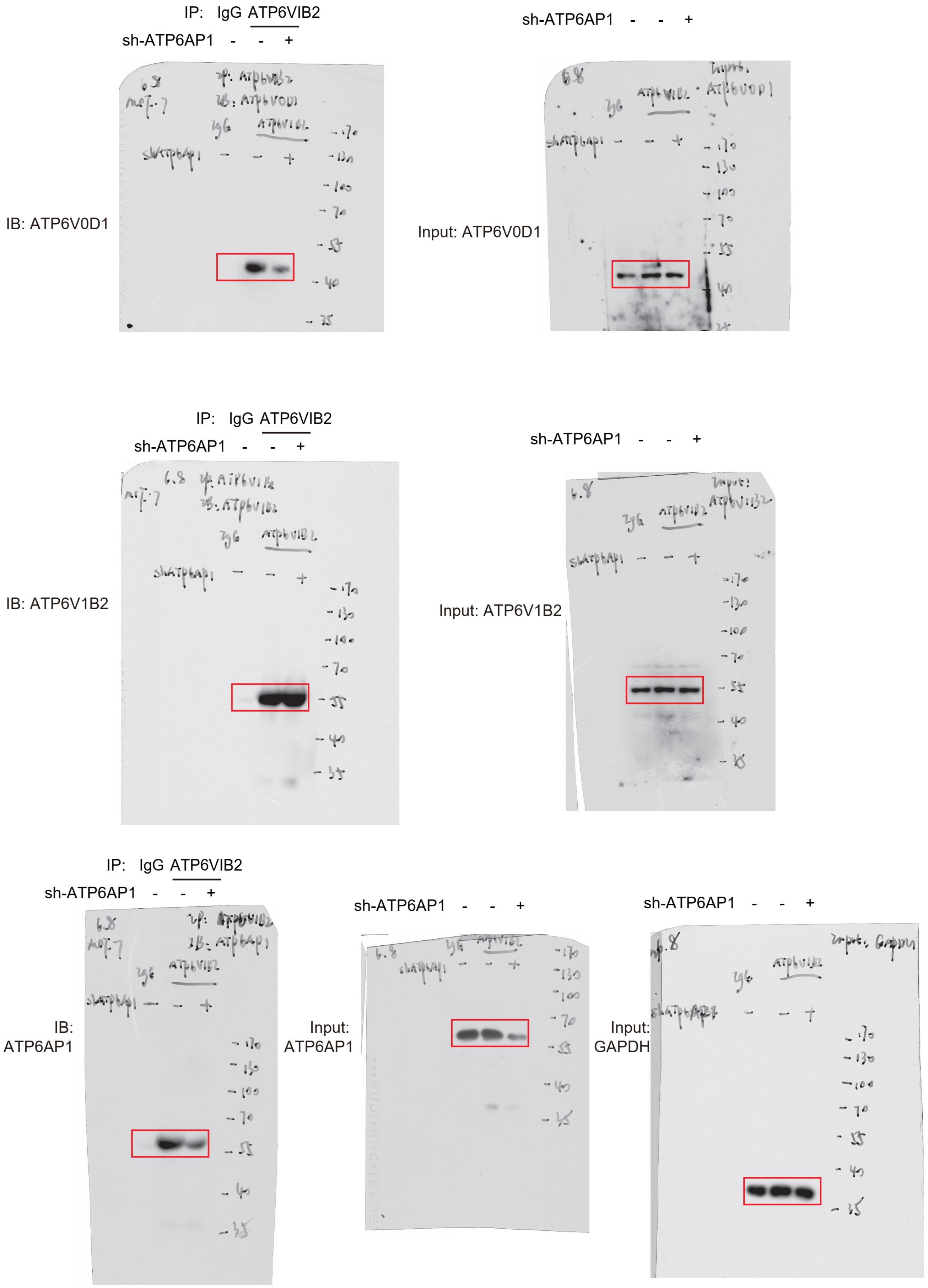
IP: IgG ATP6VIB2
sh-ATP6AP1 - - +
IB: ATP6V0D1
sh-ATP6AP1 - - +
Input: ATP6V0D1
IP: IgG ATP6VIB2
sh-ATP6AP1 - - +
IB: ATP6V1B2
sh-ATP6AP1 - - +
Input: ATP6V1B2
IP: IgG ATP6VIB2
sh-ATP6AP1 - - +
IB: ATP6AP1
sh-ATP6AP1 - - +
Input: ATP6AP1
sh-ATP6AP1 - - +
Input: GAPDH

Fig. 5C

HA-Rab7 + + +
Flag-ATP6AP1 - + -
Flag-ATP6AP2 - - +

IP: Flag
IB: HA

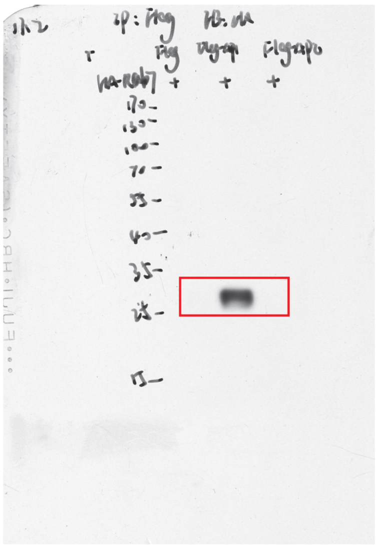

HA-Rab7 + + +
Flag-ATP6AP1 - + -
Flag-ATP6AP2 - - +

Input: HA

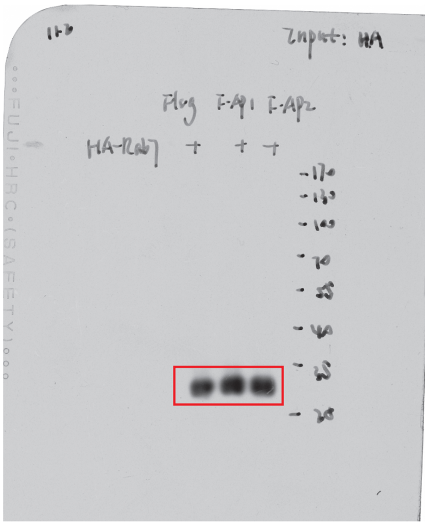

HA-Rab7 + + +
Flag-ATP6AP1 - + -
Flag-ATP6AP2 - - +

IP: Flag
IB: Flag

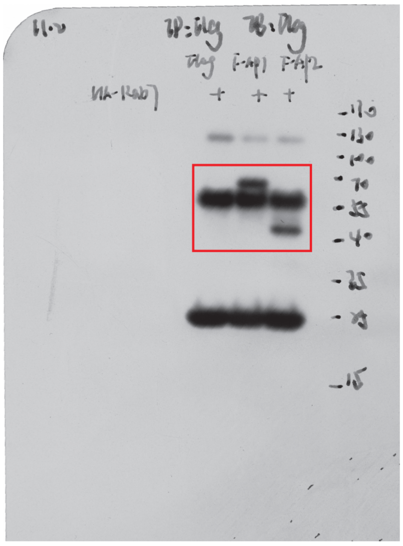

HA-Rab7 + + +
Flag-ATP6AP1 - + -
Flag-ATP6AP2 - - +

Input: Flag

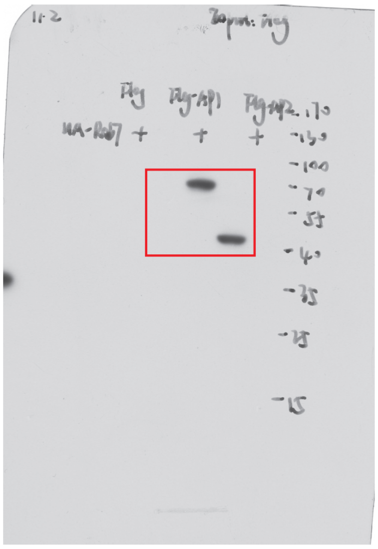

Fig. 5E

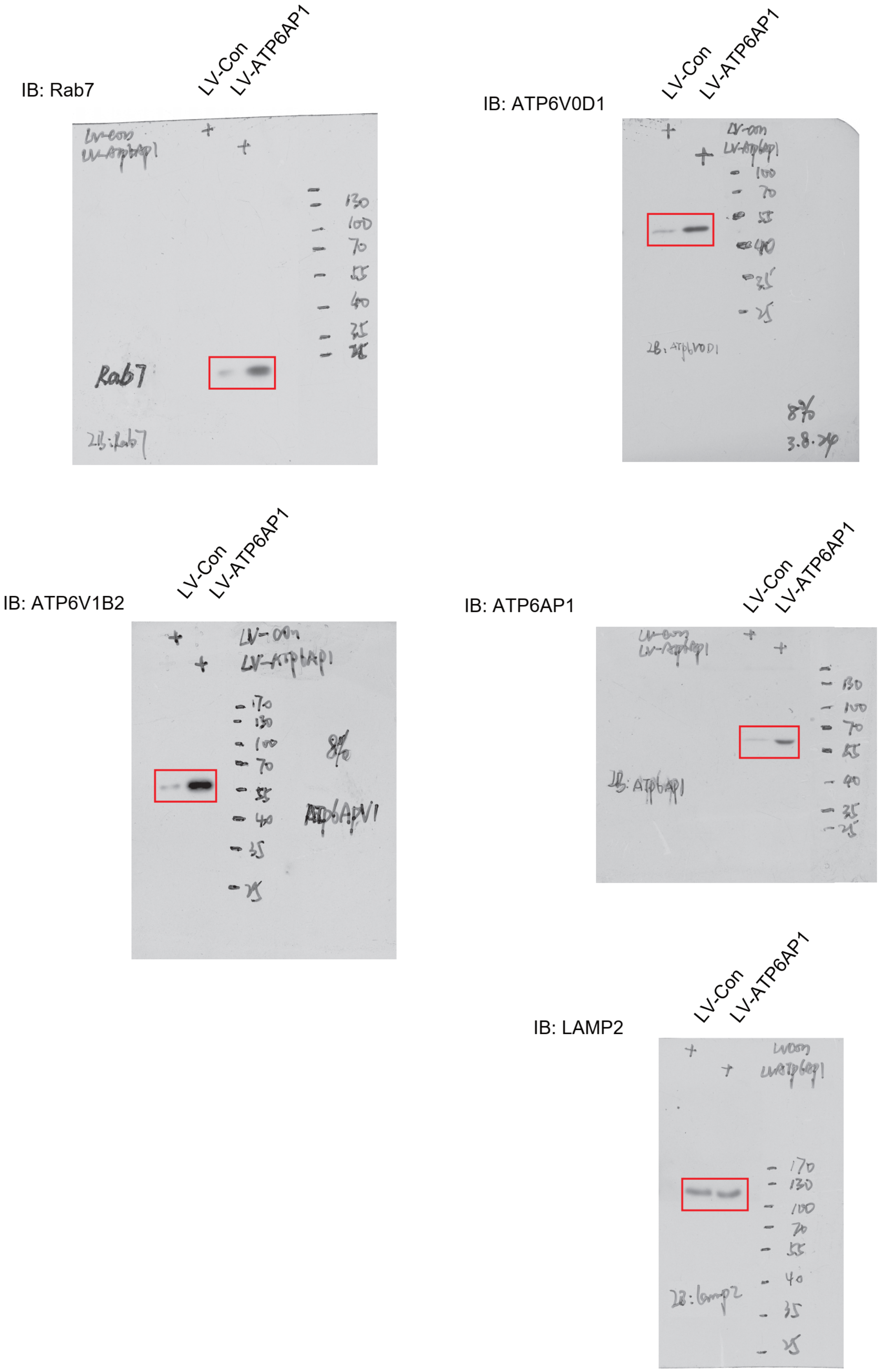
IB: Rab7
LV-Con
LV-ATP6AP1
IB: ATP6V0D1
LV-Con
LV-ATP6AP1
IB: ATP6V1B2
LV-Con
LV-ATP6AP1
IB: ATP6AP1
LV-Con
LV-ATP6AP1
IB: LAMP2
LV-Con
LV-ATP6AP1

Fig. 5F

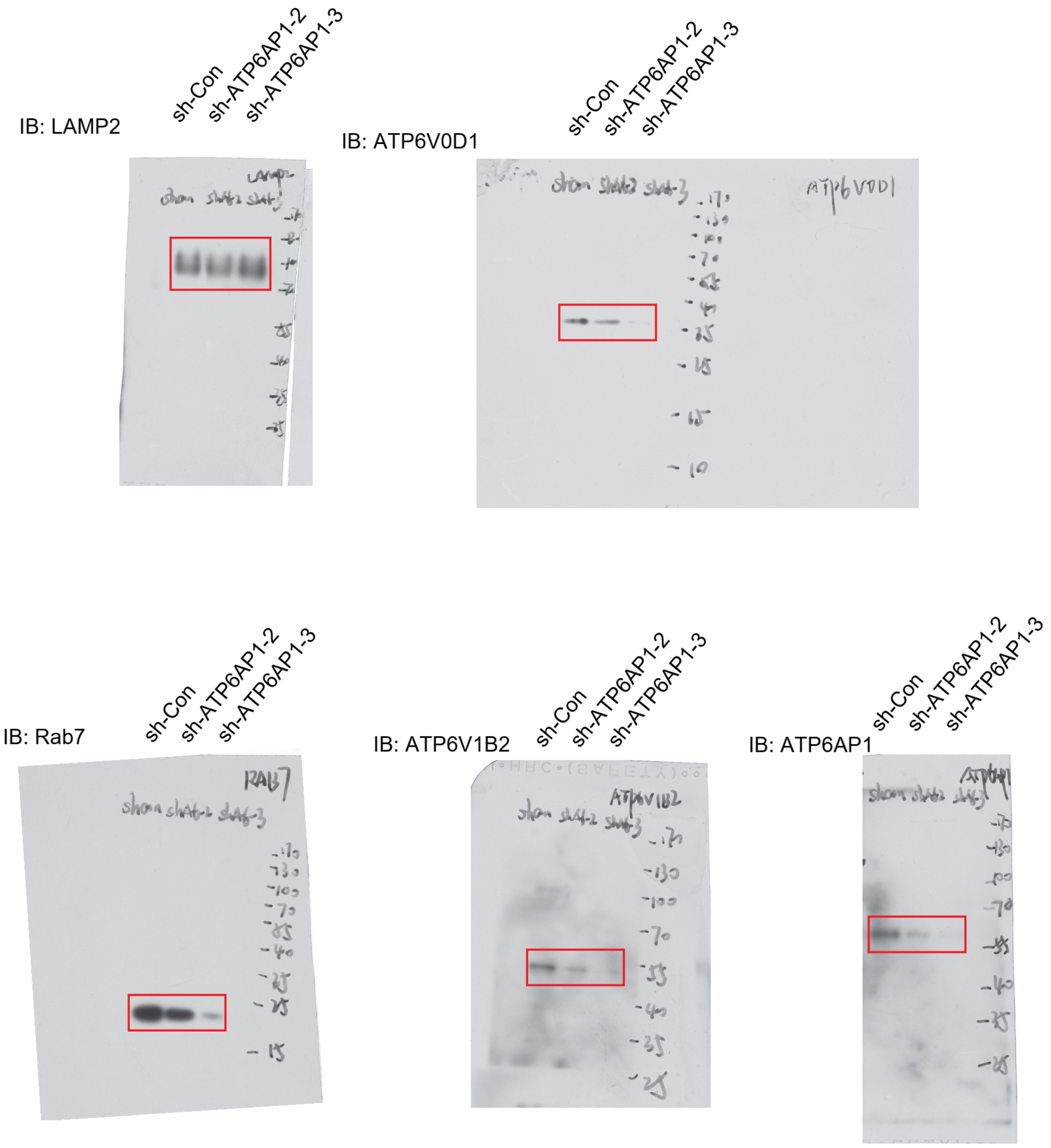
IB: LAMP2
sh-Con
sh-ATP6AP1-2
sh-ATP6AP1-3
IB: ATP6V0D1
sh-Con
sh-ATP6AP1-2
sh-ATP6AP1-3
IB: Rab7
sh-Con
sh-ATP6AP1-2
sh-ATP6AP1-3
IB: ATP6V1B2
sh-Con
sh-ATP6AP1-2
sh-ATP6AP1-3
IB: ATP6AP1
sh-Con
sh-ATP6AP1-2
sh-ATP6AP1-3

Fig. 5G

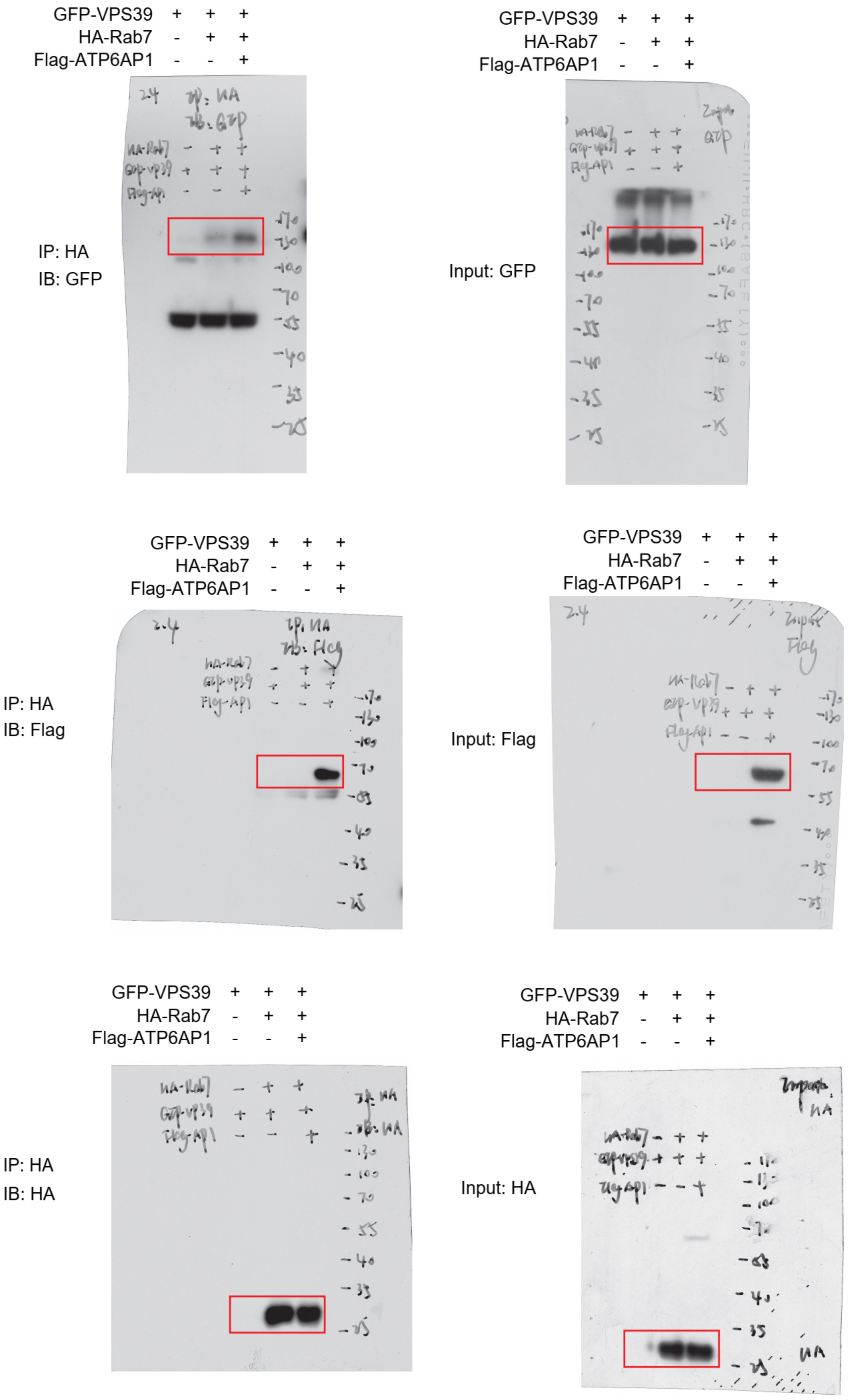
GFP-VPS39 + + +
HA-Rab7 - + +
Flag-ATP6AP1 - - +
IP: HA
IB: GFP
GFP-VPS39 + + +
HA-Rab7 - + +
Flag-ATP6AP1 - - +
Input: GFP
GFP-VPS39 + + +
HA-Rab7 - + +
Flag-ATP6AP1 - - +
IP: HA
IB: Flag
GFP-VPS39 + + +
HA-Rab7 - + +
Flag-ATP6AP1 - - +
Input: Flag
GFP-VPS39 + + +
HA-Rab7 - + +
Flag-ATP6AP1 - - +
IP: HA
IB: HA
GFP-VPS39 + + +
HA-Rab7 - + +
Flag-ATP6AP1 - - +
Input: HA

Fig. 5H

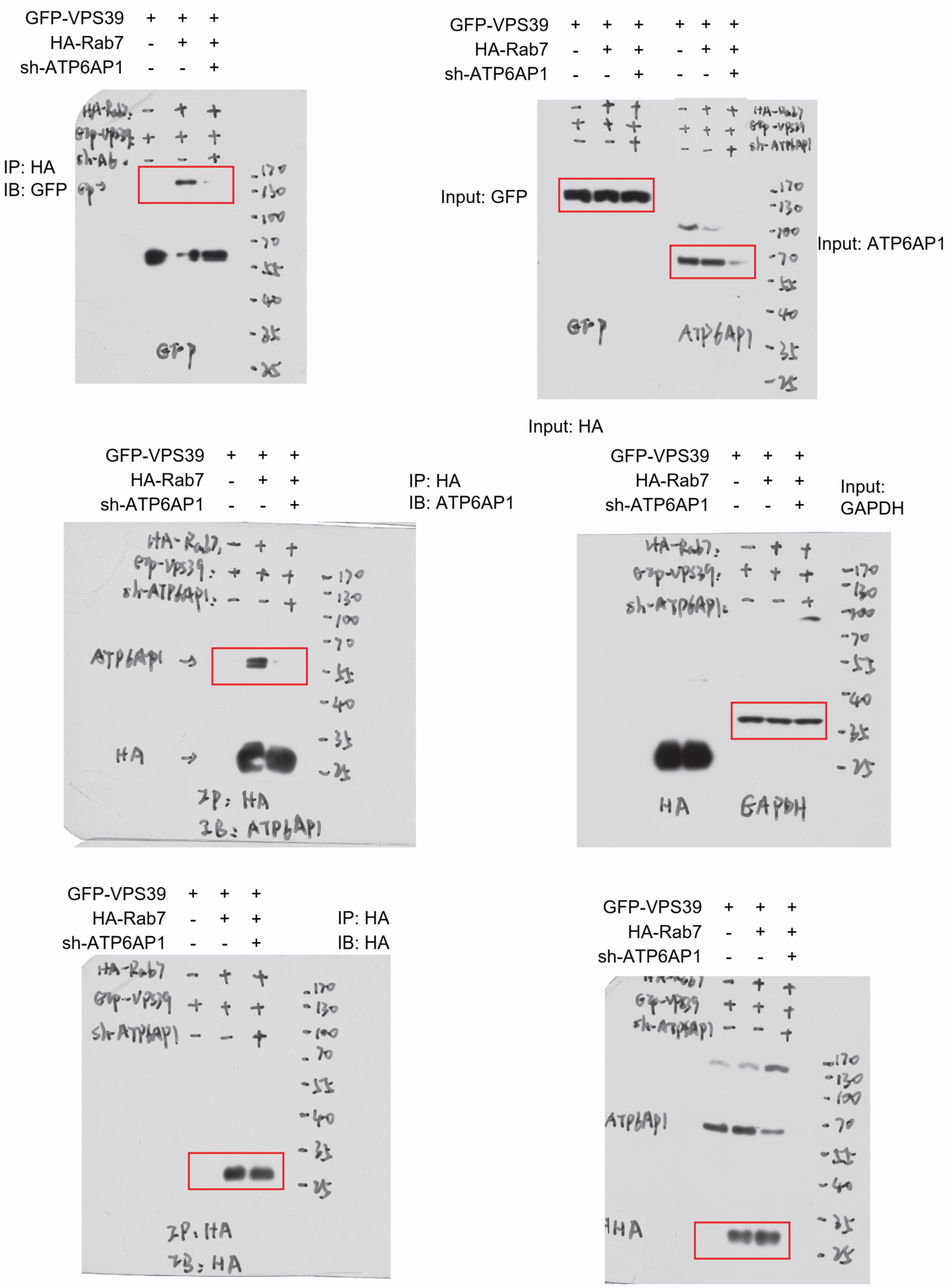
GFP-VPS39 + + +
HA-Rab7 - + +
sh-ATP6AP1 - - +
IP: HA
IB: GFP
GFP-VPS39 + + + + + +
HA-Rab7 - + + - + +
sh-ATP6AP1 - - + - - +
Input: GFP
Input: ATP6AP1
Input: HA
GFP-VPS39 + + +
HA-Rab7 - + +
sh-ATP6AP1 - - +
IP: HA
IB: ATP6AP1
GFP-VPS39 + + +
HA-Rab7 - + +
sh-ATP6AP1 - - +
Input: GAPDH
GFP-VPS39 + + +
HA-Rab7 - + +
sh-ATP6AP1 - - +
IP: HA
IB: HA
GFP-VPS39 + + +
HA-Rab7 - + +
sh-ATP6AP1 - - +

Fig. 6B

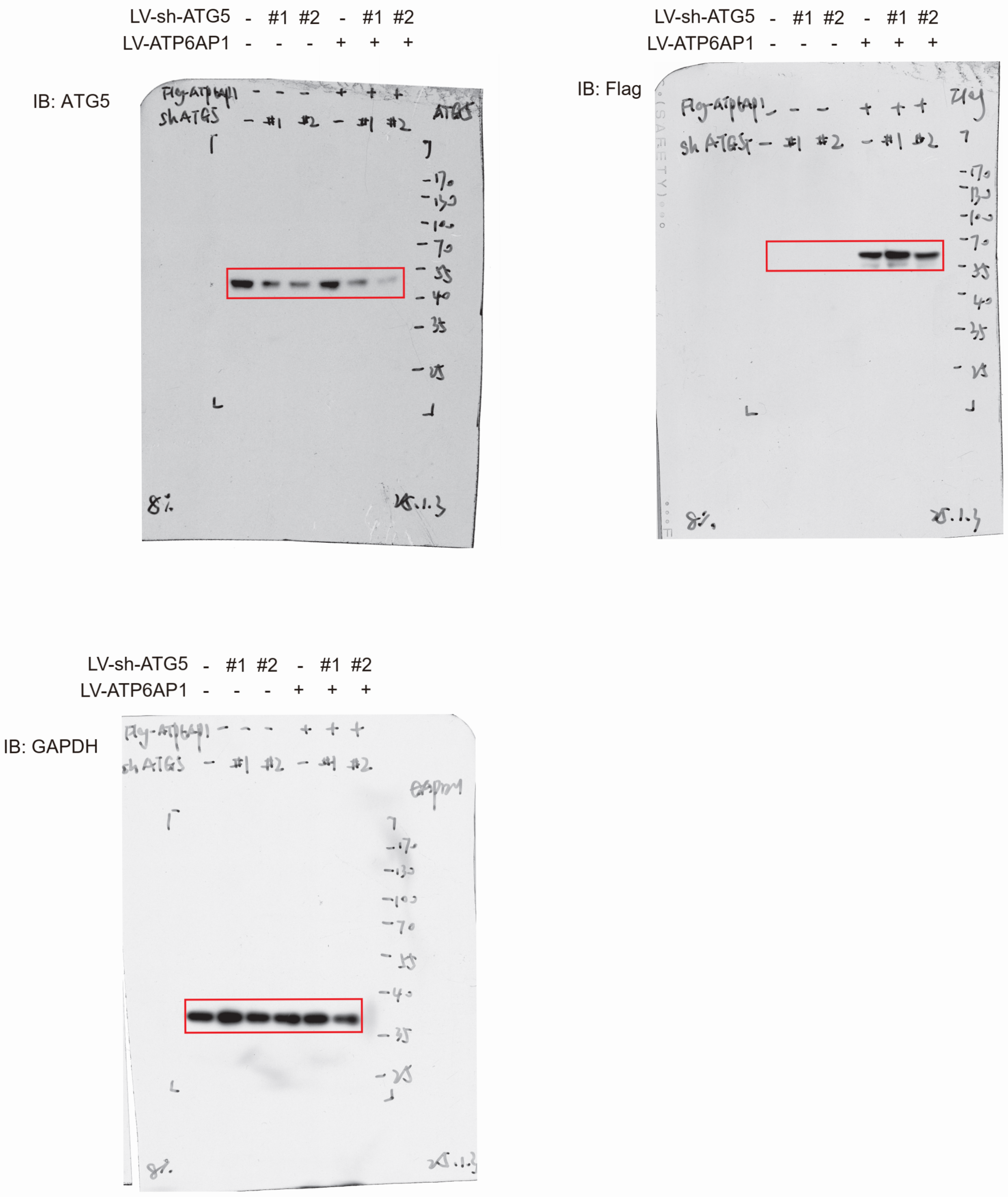
LV-sh-ATG5 - #1 #2 - #1 #2
LV-ATP6AP1 - - - + + +
IB: ATG5
LV-sh-ATG5 - #1 #2 - #1 #2
LV-ATP6AP1 - - - + + +
IB: Flag
LV-sh-ATG5 - #1 #2 - #1 #2
LV-ATP6AP1 - - - + + +
IB: GAPDH

Fig. S1B

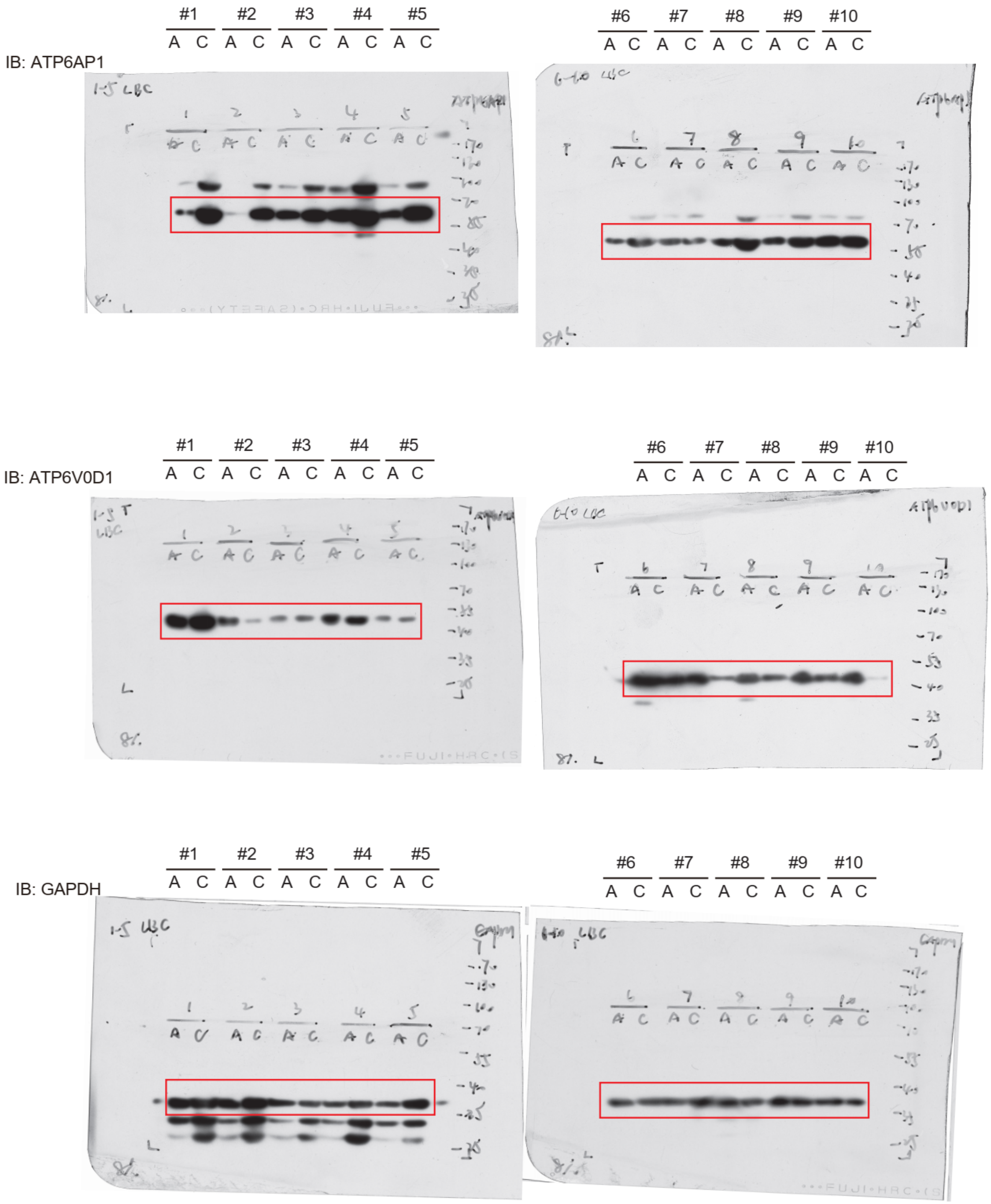
IB: ATP6AP1
#1 #2 #3 #4 #5
A C A C A C A C A C
#6 #7 #8 #9 #10
A C A C A C A C A C
IB: ATP6V0D1
#1 #2 #3 #4 #5
A C A C A C A C A C
#6 #7 #8 #9 #10
A C A C A C A C A C
IB: GAPDH
#1 #2 #3 #4 #5
A C A C A C A C A C
#6 #7 #8 #9 #10
A C A C A C A C A C

Fig. S2B

LV-ATP6AP1	-	-	-	+	+	+	-	-	-	+	+	+
Tamoxifen	-	-	-	-	-	-	+	+	+	+	+	+

IB: ATP6AP1

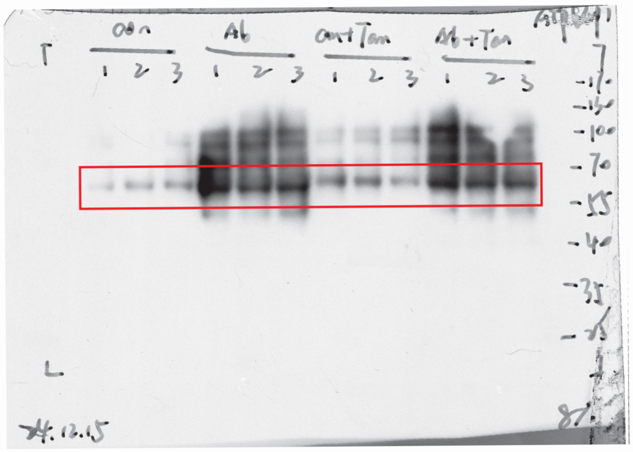

LV-ATP6AP1	-	-	-	+	+	+	-	-	-	+	+	+
Tamoxifen	-	-	-	-	-	-	+	+	+	+	+	+

IB: β-actin

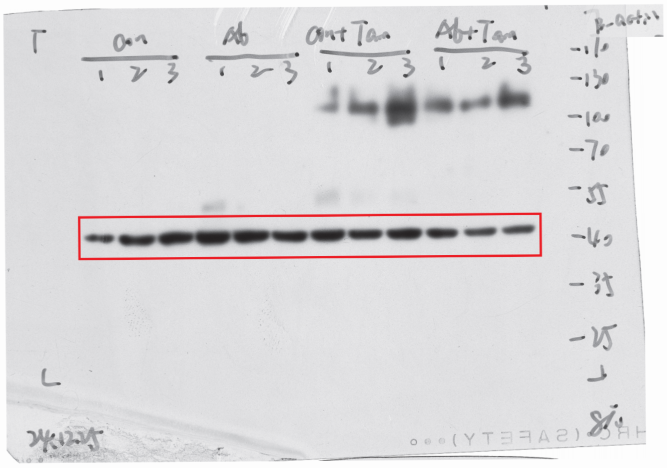

Supplement: Supplementary file 2 — Original western blots [file 41419_2025_7534_MOESM2_ESM.pdf]
